# Bulk-to-interphase polybromide confinement and electric-field-responsive recovery enable Ah-level aqueous zinc-bromine batteries at room/low temperatures

**DOI:** 10.1093/nsr/nwag393

**Published:** 2026-06-25

**Authors:** Qilong Wu, Peiyao Wang, Hong Lin, Fanxiang Meng, Chenxi Sun, Jinbao Zhao, Yang Yang

**Affiliations:** State Key Laboratory of Physical Chemistry of Solid Surfaces, State-Province Joint Engineering Laboratory of Power Source Technology for New Energy Vehicle, College of Chemistry and Chemical Engineering, Xiamen University, Xiamen 361005, China; State Key Laboratory of Physical Chemistry of Solid Surfaces, State-Province Joint Engineering Laboratory of Power Source Technology for New Energy Vehicle, College of Chemistry and Chemical Engineering, Xiamen University, Xiamen 361005, China; State Key Laboratory of Physical Chemistry of Solid Surfaces, State-Province Joint Engineering Laboratory of Power Source Technology for New Energy Vehicle, College of Chemistry and Chemical Engineering, Xiamen University, Xiamen 361005, China; State Key Laboratory of Physical Chemistry of Solid Surfaces, State-Province Joint Engineering Laboratory of Power Source Technology for New Energy Vehicle, College of Chemistry and Chemical Engineering, Xiamen University, Xiamen 361005, China; State Key Laboratory of Physical Chemistry of Solid Surfaces, State-Province Joint Engineering Laboratory of Power Source Technology for New Energy Vehicle, College of Chemistry and Chemical Engineering, Xiamen University, Xiamen 361005, China; State Key Laboratory of Physical Chemistry of Solid Surfaces, State-Province Joint Engineering Laboratory of Power Source Technology for New Energy Vehicle, College of Chemistry and Chemical Engineering, Xiamen University, Xiamen 361005, China; State Key Laboratory of Physical Chemistry of Solid Surfaces, State-Province Joint Engineering Laboratory of Power Source Technology for New Energy Vehicle, College of Chemistry and Chemical Engineering, Xiamen University, Xiamen 361005, China

**Keywords:** aqueous Zn-Br_2_ batteries, bulk-to-interphase synergistic regulation, polybromide shuttling suppression, low-temperature energy storage, high areal capacity

## Abstract

Aqueous zinc-bromine (Zn-Br_2_) batteries are promising candidates for grid-scale energy storage owing to their low cost and inherent safety. However, their areal capacity and cycling stability are severely limited by detrimental polybromide shuttling, which arises from concentration-gradient-driven diffusion and often overlooked internal-electric-field-accelerated electromigration. Herein, a bulk-to-interphase synergistic regulation strategy is proposed integrating electrostatic micropore confinement in the cathode bulk with additional interfacial anchoring and electric-field-responsive recovery at the cathode/electrolyte interphase. Poly(quaternary ammonium)-functionalized activated carbon (N^+^-AC) hosts are rationally designed to trap polybromide anions within positively charged micropores of cathode bulk, thereby suppressing concentration-gradient-driven leakage while maintaining fast conversion kinetics. Simultaneously, 1-butyl-3-methylimidazolium chloride (1,3-MI) electrolyte additive adsorbed on the cathode surface provides additional polybromide anchoring at the interphase. Importantly, theoretical calculations and *in situ* characterizations demonstrate that an electric-field-responsive 1,3-MI accumulation layer forms spontaneously during discharge, dynamically attracting polybromides and inhibiting their electromigration toward the anode, thus promoting their recovery to the cathode for reversible redox conversion. Consequently, the N^+^-AC||Zn full cell with 1,3-MI achieves stable cycling over 1000 cycles at an ultrahigh areal capacity of 10 mAh cm^−2^. Furthermore, Ah-level Zn-Br_2_ pouch cells operate stably at both room and subzero temperatures, validating the practical potential of this electric-field-aware bulk-to-interphase regulation strategy under complex climatic conditions.

## Teaser

A bulk-to-interphase regulation strategy enables full-cycle management of active bromine species, unlocking durable, high-capacity Zn-Br_2_ batteries across a wide temperature range.

## INTRODUCTION

With the rapid advancement of renewable energy technologies such as wind and solar power, the demand for electrochemical energy storage systems that combine high energy density, inherent safety and cost-effectiveness has intensified [1–8]. Aqueous zinc-bromine (Zn-Br_2_) batteries have emerged as promising candidates owing to their intrinsic merits, including high redox potential (∼1.84 V vs. Zn^2+^/Zn), abundant and low-cost zinc/bromine resources, non-flammable aqueous electrolytes and high theoretical energy density (∼440 Wh kg^−1^) [9–12]. Nevertheless, the widespread commercialization of aqueous Zn-Br static batteries remains hindered by limited achievable areal capacity and rapid performance degradation, primarily caused by the formation and shuttling of polybromide species (e.g. Br_3_^−^ and Br_5_^−^) during cycling [13–19].

Current strategies for immobilizing polybromide species can be broadly categorized into two classes. The first involves the use of porous carbon-based materials as physical hosts to confine polybromides, providing high bromine-loading capacity and conductive pathways for electron transfer [20,21]. However, the inherently weak interaction between polar polybromide anions and non-polar carbon surface severely limits immobilization efficiency, rendering this approach insufficient for long-term stable cycling [22–24]. The second strategy employs positively charged quaternary ammonium salts to chemically complex polybromide anions via strong electrostatic interactions [2,25–30]. Despite effective anchoring, this approach typically requires large quantities of quaternary ammonium salts, which significantly increase inactive mass and deteriorate electronic conductivity, compromising redox efficiency and rate performance, particularly under high loading conditions.

Importantly, polybromide-induced battery degradation is not solely a consequence of dissolution or shuttling; rather, it is intrinsically coupled to the dynamic evolution of the internal electric field during charge–discharge processes, an aspect that is often overlooked. The fundamental equilibrium governing polybromide formation is


(1) \begin{eqnarray*}
{\mathrm{B}}{{\mathrm{r}}}^ - + {\mathrm{B}}{{\mathrm{r}}}_2 \leftrightarrow {{\mathrm{Br}}}_3^ - .
\end{eqnarray*}

This equilibrium indicates that Br_3_^−^ formation requires the coexistence of sufficient Br^−^ and Br_2_. During the charging process, Br^−^ ions migrate under the internal electric field and accumulate at the cathode, where they are oxidized to Br_2_. At the cathode/electrolyte interphase, a portion of generated Br_2_ reacts with locally enriched Br^−^ to form Br_3_^−^, explaining why polybromide formation is predominantly observed during charging [14,26]. The transport behavior of Br_3_^−^ from the cathode interphase into the bulk electrolyte (*J*) is governed by two fluxes: diffusion driven by concentration gradients (*J*_d_) and electromigration driven by electric field (*J*_m_):


(2) \begin{eqnarray*}
{J}_{\mathrm{d}} = - D\frac{{\partial {C}_{\left( x \right)}}}{{\partial x}},
\end{eqnarray*}

(3) \begin{eqnarray*}
{J}_{{\mathrm{m}}\ } = \ - \frac{{zF}}{{RT}}DC\frac{{\partial {\phi}_{( x )}}}{{\partial x}},
\end{eqnarray*}

(4) \begin{eqnarray*}
J = {J}_{\mathrm{d}} + \ {J}_{\mathrm{m}} = - D\frac{{\partial {C}_{\left( x \right)}}}{{\partial x}} - \frac{{zF}}{{RT}}DC\frac{{\partial {\phi}_{( x )}}}{{\partial x}},
\end{eqnarray*}

where *J* is the ionic flux, *D* is the diffusion coefficient, *R* is the gas constant, *F* is the Faraday constant and *z* is the charge number; ∂*C*_(_*_x_*_)_/∂*x* and ∂*ϕ*(*x*)/∂*x* represent the concentration and potential gradient, respectively. During charging, diffusion tends to push Br_3_^−^ away from the cathode, but this flux is partially counteracted by oppositely directed electromigration, leading to substantial Br_3_^−^ accumulation at the cathode/electrolyte interphase and only minimal leakage into the bulk electrolyte (Fig. 1a). However, upon discharge, the internal electric field reverses. Although a fraction of interfacial polybromide species can be directly reduced back to Br^−^, and thus reutilized in the redox cycle, another fraction is driven outward by the combined effects of diffusion and the reversed electric field. This co-driven transport promotes directional Br_3_^−^ migration toward the zinc anode, where parasitic redox reactions induce severe Zn corrosion and pronounced self-discharge (Fig. 1b). Therefore, while enhancing bromine affinity and redox efficiency within the bulk cathode can mitigate concentration-gradient-driven leakage, a more critical and less-explored strategy is to actively inhibit the electric-field-driven electromigration of polybromide anions during discharge.

**Figure 1.**
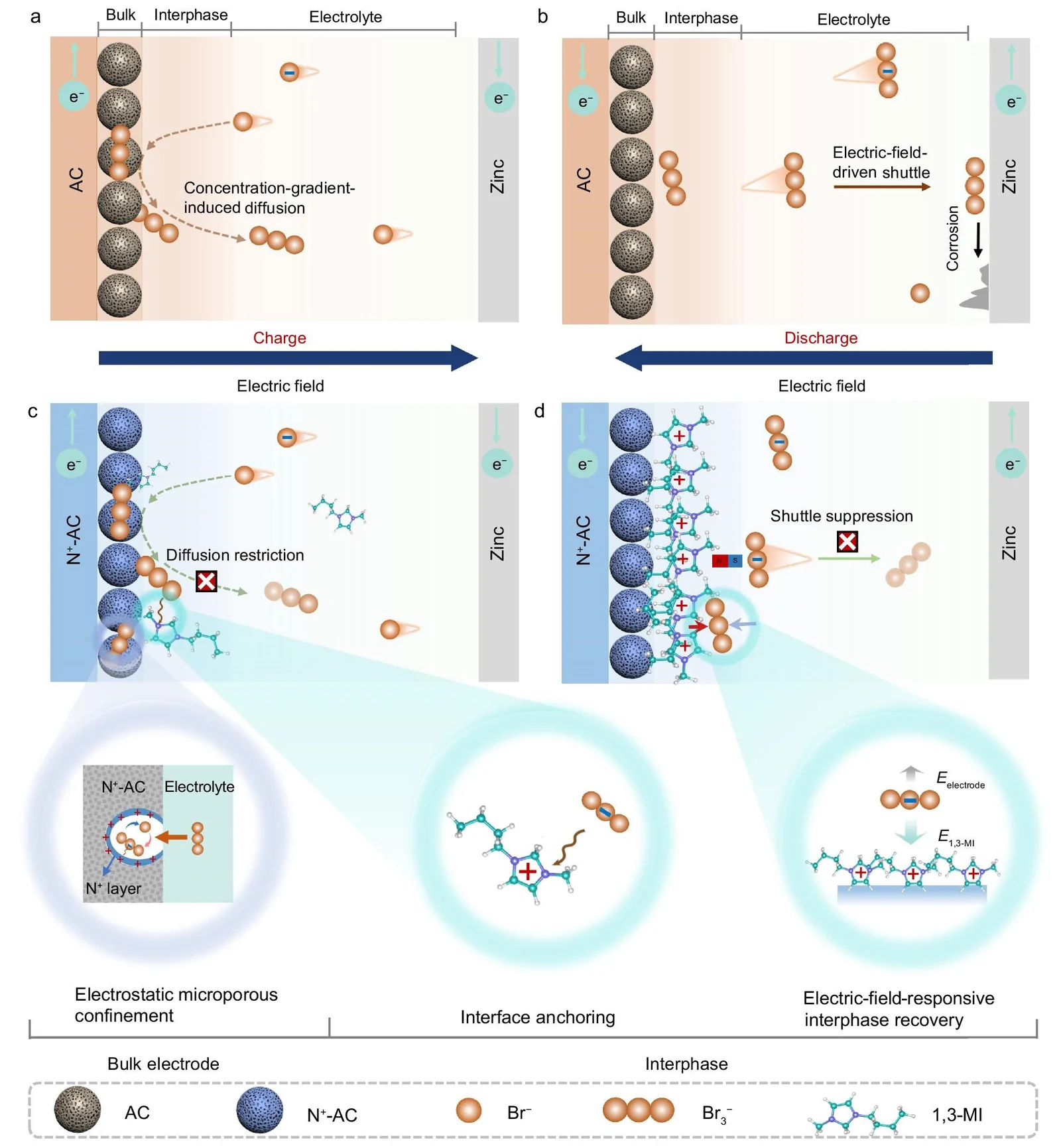
Schematic illustration of (a) polybromide generation and diffusion during charging, (b) electric-field-driven polybromide migration during discharging and the bulk-to-interphase synergistic regulation mechanism for suppressing polybromide shuttling during (c) charge and (d) discharge.

In this work, we develop a bulk-to-interphase synergistic regulation strategy to suppress polybromide dissolution and shuttling throughout the entire charge–discharge process by integrating electrostatic micropore confinement in the cathode bulk with interface anchoring and electric-field-responsive recovery at the cathode/electrolyte interphase. Specifically, activated carbon (AC) is functionalized with poly(diallyldimethylammonium chloride) to introduce abundant cationic sites within its micropores (N^+^-AC). This modification strengthens electrostatic confinement of polybromide species inside AC micropores, thereby suppressing their diffusion from the cathode bulk to the electrolyte interface during charging. Meanwhile, 1-butyl-3-methylimidazolium chloride (1,3-MI) is adopted as an electrolyte additive to provide additional polybromide anchoring at the interface via strong electrostatic interactions, effectively capturing polybromide species that escape the cathode bulk (Fig. 1c). More importantly, during discharge, 1,3-MI cations accumulate at the cathode/electrolyte interphase following the internal electric field direction, forming a positively charged interfacial layer. This field-assembled layer dynamically attracts polybromide species and inhibits their electric-field-driven migration toward the anode, thereby promoting their recovery to the cathode for reversible redox conversion (Fig. 1d). Consequently, this bulk-to-interphase synergistic regulation enables the Zn-Br_2_ battery to achieve a high areal capacity of 10 mAh cm^−2^ over 1000 cycles. Furthermore, owing to the fast redox kinetics and intrinsic low-temperature tolerance of the ZnBr_2_-based electrolyte, this strategy is further extended to Ah-level pouch cells that operate reliably at subzero temperatures (−20°C). This study highlights the importance of targeted, electric-field-aware interphase manipulation in mitigating polybromide shuttling across the full charge–discharge cycle, providing practical guidelines for high-areal-capacity aqueous Zn-Br_2_ batteries.

## RESULTS AND DISCUSSION

### Theoretical validation for suppressing polybromide leaching

To suppress polybromide diffusion from the cathode bulk to the electrode/electrolyte interphase, high-specific-surface-area AC (2286 m^2^ g^−1^; Fig. S1) was functionalized with poly(diallyldimethylammonium chloride) (denoted as N^+^) and fabricated into a dry-processed cathode (Fig. S2) to realize high areal capacity [31]. As illustrated in Fig. 2a, the resulting self-standing N^+^-AC electrode exhibits good mechanical robustness and can be manufactured as a continuous electrode roll, indicating compatibility with scalable production. Tensile testing further verifies this merit, with a maximum elongation of over 100% and a gradually increased stress up to 0.12 MPa (Fig. S3). As the N^+^ concentration in the treatment solution increases from 0 wt% to 1 wt%, the zeta potential of AC shifts from −34.9 mV to +48.8 mV (Fig. 2b), reversing the surface charge nature to enable electrostatic confinement of polybromide anions. Considering that excessive N^+^ content reduces the specific surface area of AC (Fig. S4) and can compromise adsorption capability, the treatment concentration was finally optimized to be 0.05 wt%, corresponding to a N^+^ loading of ∼3.9 wt% in N^+^-AC with a considerable positive zeta potential of 36.2 mV (Fig. S5). Meanwhile, the electronic conductivity of N^+^-AC (1.52 S cm^−1^) remains comparable to that of pristine AC (1.65 S cm^−1^) (Table S1). Elemental mapping reveals homogeneous distributions of N and Cl, confirming uniform incorporation of N^+^ species (Fig. S6). Notably, no N^+^-related signals are detected in the Raman spectra of N^+^-AC (Fig. S7), suggesting that N^+^ species are not present as surface-exposed aggregates. In addition, ultraviolet-visible (UV-vis) spectra of the supernatant collected after soaking N^+^-AC in water for 12 h show no discernible N^+^-associated absorption peaks, whereas the recovered N^+^-AC powder after drying retains a strongly positive zeta potential of +32.3 mV (Figs S8 and S9). Collectively, these results indicate that N^+^ species are predominantly fixed within the microporous AC framework and exhibit negligible solubility in aqueous media.

**Figure 2.**
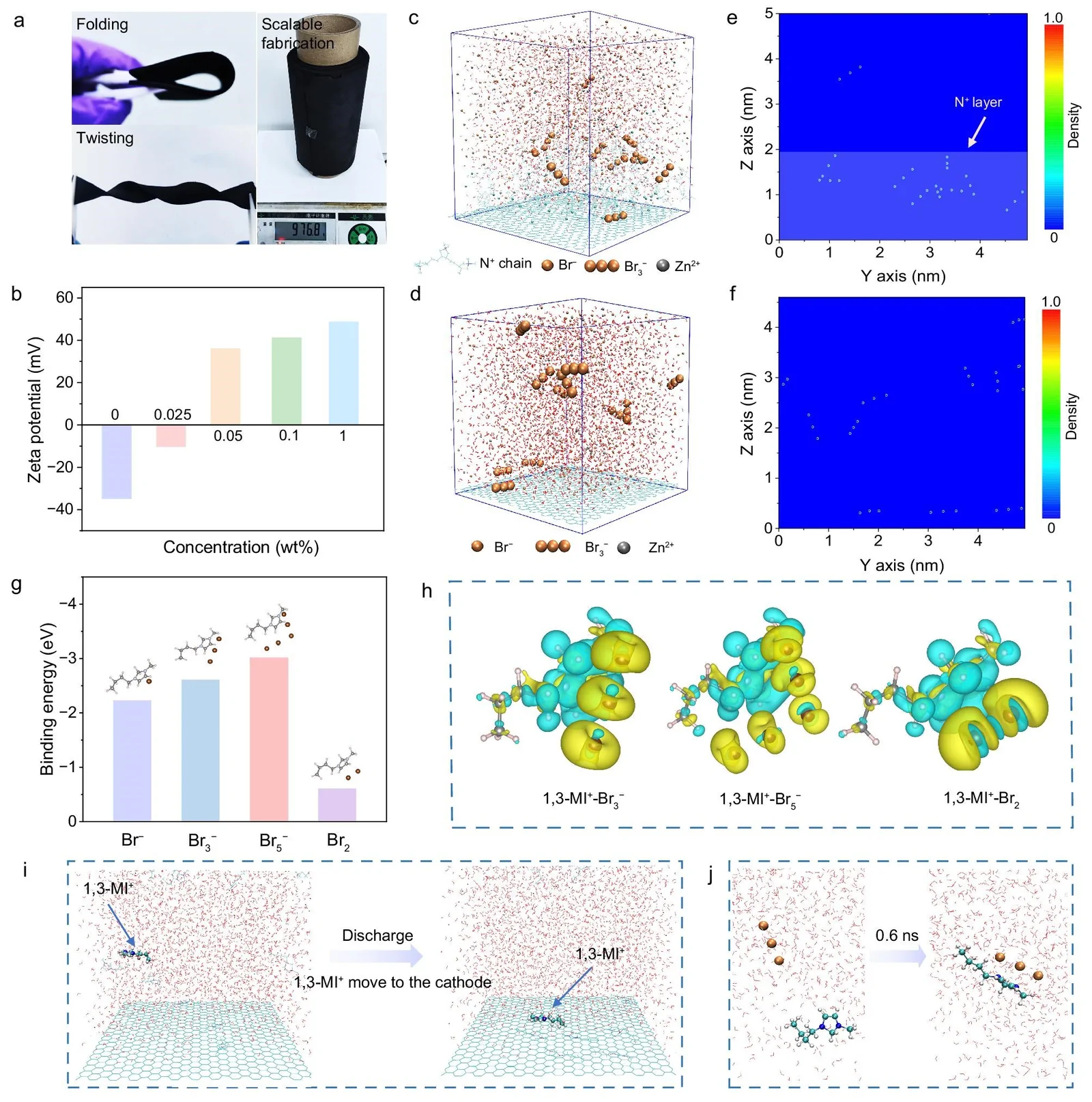
(a) Optical images of dry electrodes in different states. (b) Zeta potentials of AC treated with N^+^ solutions at different concentrations. MD simulations of (c) N^+^-AC and (d) AC in the Br_3_^−^ and 3 m ZnBr_2_ mixed solution, and (e, f) corresponding density distribution map of Br_3_^−^ anions. (g) Binding energies of 1,3-MI with Br^−^, Br_3_^−^, Br_5_^−^ and Br_2_. (h) The differential charge density diagrams of 1,3-MI with Br_3_^−^, Br_5_^−^ and Br_2_ (yellow and blue represent electron accumulation and electron depletion, respectively). (i) MD simulations of the 1,3-MI electrolyte during discharge. (j) A free Br_3_^−^ anion and a 1,3-MI^+^ cation gradually approach each other spontaneously.

To validate the polybromide-trapping ability of N^+^-AC, theoretical calculations were initially carried out. Molecular dynamics (MD) simulations show that polybromide anions preferentially accumulate at the N^+^-containing interface relative to the N^+^-free counterpart (Fig. 2c–f), and the strongly negative binding energies between N^+^ molecules and polybromides further confirm their robust interactions (Fig. S10). Accordingly, N^+^ functionalization strengthens the micropore confinement effect of AC and suppresses polybromide leaching from the cathode bulk to the cathode/electrolyte interphase during charge. This enhanced polybromide adsorption capability is further supported by a straightforward adsorption experiment (Fig. S11). Nevertheless, under high areal capacity conditions (>5 mAh cm^−2^), completely preventing polybromide escape from the cathode over prolonged cycling is hard to achieve. We therefore introduce 1,3-MI as an electrolyte additive to anchor polybromide anions at the interphase, suppress their diffusion into the bulk electrolyte and thereby achieve synergistic inhibition of polybromide shuttling. Simultaneously, the introduction of 1,3-MI also decreases the charge transfer resistance, further enhancing electrochemical kinetics (Fig. S12). A simple adsorption test confirms that 1,3-MI can coordinate with polybromides to form an oily, insoluble complex (Fig. S13). Furthermore, the calculated binding energies of 1,3-MI with Br^−^, Br_3_^−^, Br_5_^−^ and Br_2_ are −2.2, −2.6, −3.0 and −0.61 eV, respectively (Fig. 2g). Charge density difference maps for 1,3-MI complexed with Br_3_^−^, Br_5_^−^ and Br_2_ further visualize the intensive charge redistribution upon binding (Fig. 2h), corroborating the interaction between 1,3-MI and bromine species. Notably, the binding strength of the small-molecule 1,3-MI toward polybromides is weaker than that of the polymeric N^+^ molecule in the N^+^-AC cathode, which is advantageous for dynamic adsorption/desorption and polybromide reutilization during cycling. Moreover, MD simulations reveal the migration and enrichment of 1,3-MI toward the cathode during discharge (Fig. 2i). In addition, spontaneous approaching between a free 1,3-MI^+^ ion and a free Br_3_^−^ ion in the electrolyte is observed within 0.6 ns (Fig. 2j), further supporting its favorable affinity for polybromides. Collectively, these results indicate that 1,3-MI can spontaneously anchor polybromides at the cathode interphase, thereby effectively mitigating their outward diffusion into the bulk electrolyte.

### Electrochemical performance at room temperature

To verify the efficacy of the bulk-to-interphase synergistic regulation strategy in Zn-Br_2_ batteries, comprehensive electrochemical performance tests were conducted at room temperature. Cyclic voltammetry (CV) measurements were first performed on AC||Zn, N^+^-AC||Zn and N^+^-AC||Zn cells with 1,3-MI within a voltage window of 0.5–1.9 V at 1 mV s^−1^ (Fig. S14). The AC||Zn and N^+^-AC||Zn cells show similar oxidation behavior, indicating unaffected electron/ion transport upon N^+^ modification. During the reduction process, N^+^-AC||Zn cell delivers a larger peak area and lower polarization compared to AC||Zn cell, suggesting improved bromide/polybromide conversion reversibility. Notably, further incorporation of 1,3-MI leads to the highest peak current response and the lowest polarization, indicative of accelerated redox kinetics and suppressed polybromide shuttling. Consistent with the CV results, as shown in Fig. 3a, the N^+^-AC||Zn cell delivers discharge areal capacities of 9.3 mAh cm^−2^ at 5 mA cm^−2^ and 9.7 mAh cm^−2^ at 50 mA cm^−2^, both higher than those of the bare AC||Zn cell (9.0 and 9.4 mAh cm^−2^, respectively). Upon introducing 1,3-MI into the electrolyte, the discharge capacities of the N^+^-AC||Zn cell are further improved to 9.7 mAh cm^−2^ at 5 mA cm^−2^ and 9.9 mAh cm^−2^ at 50 mA cm^−2^. As demonstrated in Fig. 3b, the optimized N^+^-AC||Zn cell with 1,3-MI achieves a high average Coulombic efficiency (ACE) of 98.6% over 1000 cycles for a high areal capacity of 10 mAh cm^−2^ at 20 mA cm^−2^. In contrast, the N^+^-AC||Zn cell remains stable for 181 cycles with a slightly lower ACE of 96.0%, whereas the unmodified AC||Zn cell fails after only 30 cycles, accompanied by a pronounced drop in ACE to 94.3%. Even at an ultrahigh current density of 50 mA cm^−2^, the N^+^-AC||Zn cell with 1,3-MI still maintains stable cycling for over 800 cycles (Fig. S15). As summarized in Fig. 3c and Table S2, this electrochemical performance surpasses most previously reported static Zn-Br_2_ battery systems in terms of combined areal capacity and cycling durability [1,3,4,11,25,32–39]. To further enhance the energy density, we also assembled an anode-free configuration (N^+^-AC||Ti) with 1,3-MI. This cell can also sustain long-term cycling for >2000 cycles with a considerable ACE of 96.7% at 10 mAh cm^−2^ (Fig. 3d and Fig. S16), indicating robust reversibility even under Zn-free conditions. In addition, self-discharge behavior was also assessed by resting the cells for 6 h after charging to 10 mAh cm^−2^. As presented in Fig. 3e, the N^+^-AC||Zn cell with 1,3-MI achieves a markedly higher capacity retention of 83.9%, compared with 65.4% for N^+^-AC||Zn and 60.6% for AC||Zn cells, confirming effective suppression of the polybromide shuttle in the optimized system. To demonstrate practical viability, a large-format (10 cm × 10 cm) N^+^-AC||Zn pouch cell with 1,3-MI corresponding to a cell-level capacity of ∼1 Ah was fabricated. It exhibits stable cycling over 145 cycles with a high CE of 97.0%. To the best of our knowledge, this is the first reported Ah-level Zn-Br_2_ pouch cell to achieve operation exceeding 140 cycles (Fig. 3f and Table S3) [1–4,11,25,34,38–40]. Impressively, the 1 Ah pouch cell exhibits no smoke or fire during nail penetration tests (Fig. S17). Moreover, a smaller pouch cell (3 cm × 3 cm) remains capable of lighting a 2.0 V LED after bending, cutting and needle penetration, highlighting outstanding mechanical tolerance and safety (Fig. 3g). Notably, three pouch cells connected in series provide a stacked voltage as high as 5.1 V and can power a toy car traveling over 478 m after charging to 45 mAh (Fig. 3h).

**Figure 3.**
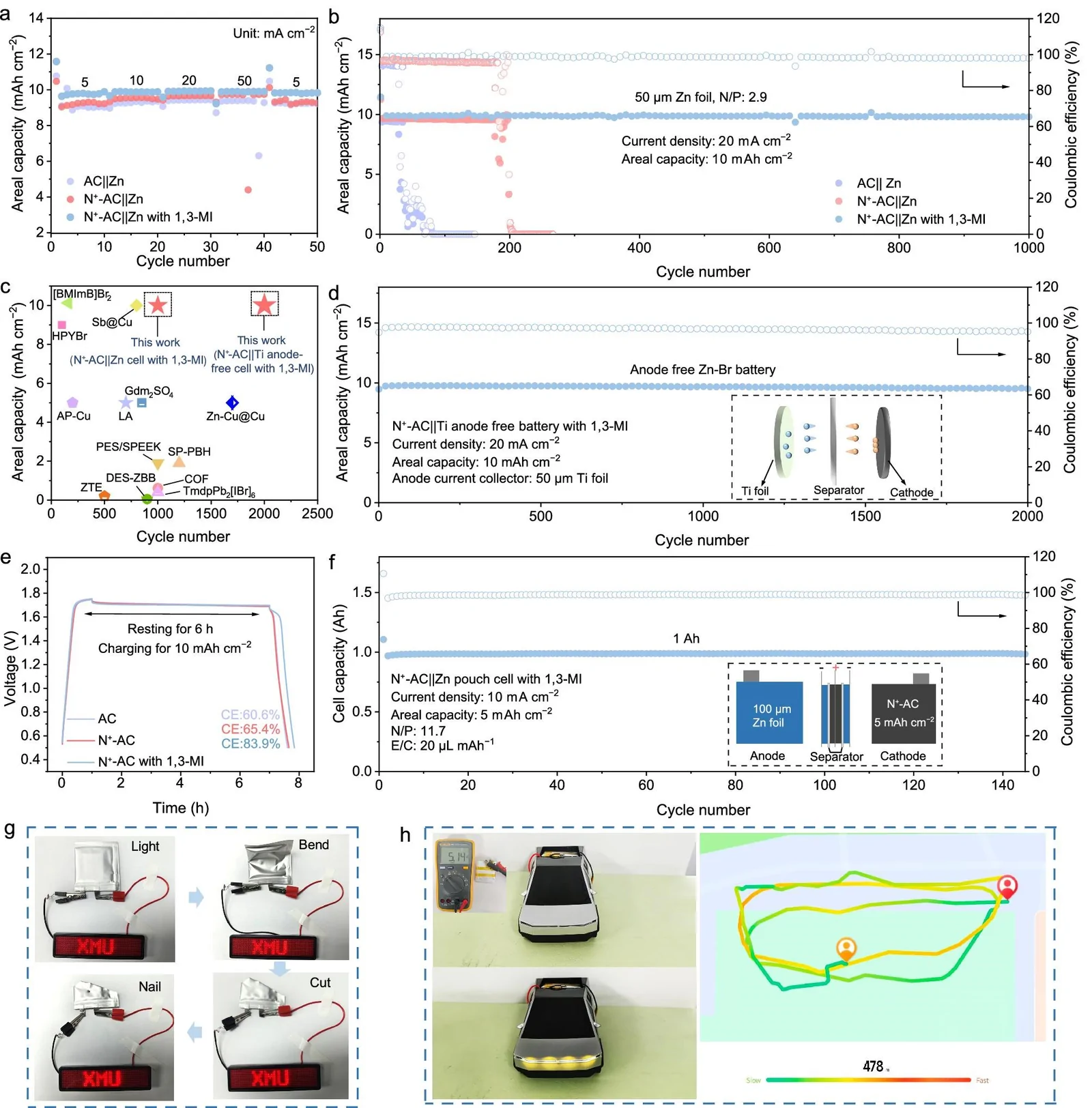
Electrochemical performance of ZBBs at room temperature. (a) Rate capability of AC||Zn, N^+^-AC||Zn and N^+^-AC||Zn cells with 1,3-MI at a constant charge areal capacity of 10 mAh cm^−2^ and discharge to 0.5 V. (b) Cycling performance of AC||Zn, N^+^-AC||Zn and N^+^-AC||Zn cells with 1,3-MI (50 µm Zn foil) at 10 mAh cm^−2^ and 20 mA cm^−2^. (c) Comparison of the work with previously reported Zn-Br_2_ batteries. (d) Cycling performance of N^+^-AC||Ti anode-free cell with 1,3-MI at 10 mAh cm^−2^ and 20 mA cm^−2^. (e) Self-discharge time–voltage profiles of AC||Zn, N^+^-AC||Zn and N^+^-AC||Zn cells with 1,3-MI. (f) Cycling performance of N^+^-AC||Zn pouch cell with 1,3-MI (the inset is the configuration of the pouch cell). Images of pouch cells (g) powering 2.0 V LED lights and (h) driving trajectories of toy cars propelled by series-connected pouch cells.

### Mechanistic investigation of polybromide shuttle suppression

The remarkable electrochemical improvement described above originates from effective suppression of polybromide shuttling throughout the full charge–discharge process. To elucidate this mechanism, *in situ* UV-vis and *in situ* Raman spectroscopy were performed (Fig. 4a and Fig. S18). For the bare AC||Zn cell, intensified polybromide absorption bands (∼244–349 nm) appear in the UV-vis spectra during the cycling (Fig. 4b). In contrast, these signals weaken in the N^+^-AC||Zn cell and are further minimized in the N^+^-AC||Zn cell with 1,3-MI (Fig. 4c and d), indicating stepwise suppression of polybromide dissolution and diffusion along its transport pathway. This conclusion is further supported by *in situ* Raman measurements using a home-made electrochemical cell. Distinct characteristic peaks of Br_5_^−^ at ∼257 cm^−1^ emerge during charging for the AC||Zn cell (Fig. 4e and f), decrease substantially for the N^+^-AC||Zn cell (Fig. 4g) and become nearly undetectable for the N^+^-AC||Zn cell with 1,3-MI (Fig. 4h). Collectively, these spectroscopic results provide direct evidence that the proposed bulk-to-interphase synergistic regulation strategy effectively mitigates polybromide shuttling by simultaneously manipulating dissolution and diffusion processes.

**Figure 4.**
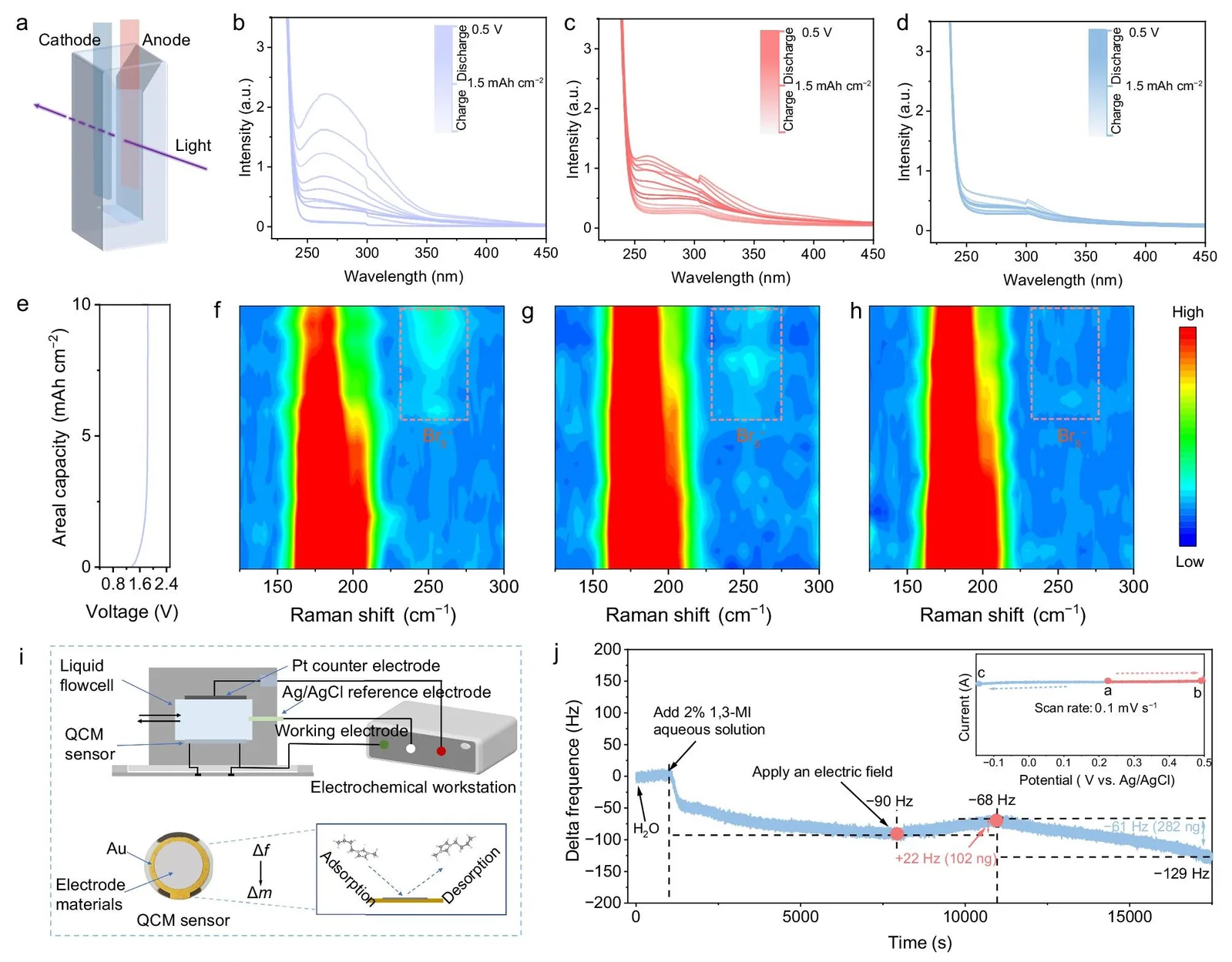
Inhibition mechanism of polybromide shuttling. (a) Optical images of quartz cells for *in situ* UV-vis spectroscopy measurements. (b–d) *In situ* UV-vis spectra of AC||Zn, N^+^-AC||Zn and N^+^-AC||Zn cells with 1,3-MI during charge–discharge process. *In situ* Raman spectra of (f) AC||Zn, (g) N^+^-AC||Zn and (h) N^+^-AC||Zn cells with 1,3-MI, and (e) corresponding charging curve. (i) Schematic diagram of EQCM device and (j) EQCM measurement of 2 wt% 1,3-MI aqueous solution (without ZnBr_2_), with the inset showing the CV curve scanned anodically to 0.5 V and cathodically to −0.15 V.

To further probe the electric-field-responsive adsorption/desorption behavior of 1,3-MI at the cathode/electrolyte interface, electrochemical quartz crystal microbalance (EQCM) measurements were performed (Fig. 4i). As shown in Fig. 4j, applying an anodic electric field (scan rate of 0.1 mV s^−1^; inset in Fig. 4j) induces a gradual frequency increase of 22 Hz, corresponding to an apparent mass loss of 102 ng. In contrast, cathodic scanning causes a 61 Hz frequency reduction and a 282 ng apparent mass gain, revealing distinct potential-governed interfacial mass evolution. To exclude solvent contributions, blank EQCM tests conducted in pure water reveal only minimal frequency shifts (−3.2 Hz) under cathodic polarization, verifying that solvent-side interfacial effects are negligible (Fig. S19). In comparison, the pronounced, potential-dependent response in 1,3-MI solution confirms that the observed frequency–mass variations originate from specific interfacial interactions of 1,3-MI. Accordingly, the anodic mass loss and cathodic mass gain are primarily ascribed to desorption and electric-field-driven adsorption/accumulation of 1,3-MI, respectively, accompanied by partial displacement of interfacial water molecules. Notably, the final frequency remains much lower than the initial value before polarization, directly confirming considerable net accumulation of 1,3-MI at the electrode/electrolyte interphase during discharge under practical full-cell conditions.

### Electrolyte anti-freezing mechanism and electrochemical performance at −20°C

Electrochemical energy-storage systems must tolerate diverse climatic conditions, and low-temperature stability is therefore a key metric for practical deployment. Accordingly, the low-temperature applicability of this system was further evaluated, with an emphasis on the anti-freezing capability of the electrolyte. Differential scanning calorimetry (DSC, Fig. 5a) shows that both the 3 m ZnBr_2_ electrolyte and 3 m ZnBr_2_ + 2 wt% 1,3-MI electrolyte exhibit ultralow freezing points of −36.8°C and −35.9°C, respectively, indicating that the anti-freezing behavior is primarily governed by the ZnBr_2_ salt. To elucidate the mechanism, the O–H microenvironment of water was probed by Raman and nuclear magnetic resonance (NMR) spectroscopy. As depicted in Fig. S20, adding 3 m ZnBr_2_ to water induces a clear blue shift of the broad O–H stretching band (∼3000–3800 cm^−1^). Deconvolution results reveal a decreased fraction of hydrogen-bonded water (strong + weak) and an increased fraction of non-hydrogen-bonded water, evidencing disruption of the native H-bond network and thus suppressing freezing. Consistently, the ^1^H NMR signal of water also shifts upfield upon ZnBr_2_ addition (Fig. S21), indicative of enhanced proton shielding and increased electron density around hydrogen nuclei, further supporting weakened hydrogen bonding. To rationalize these observations, theoretical calculations were performed. As shown in Fig. 5b, the binding energies of H_2_O···Br^−^ and H_2_O···Zn^2+^ are significantly higher than that of H_2_O···H_2_O, favoring ion–water interactions and destabilizing intermolecular water–water hydrogen bonds. MD simulations further confirm a reduced total number of hydrogen bonds in the presence of 3 m ZnBr_2_ (Fig. 5c), consistent with disruption of the water H-bond network. In the 3 m ZnBr_2_ electrolyte, populations associated with H_2_O–H_2_O, H_2_O–Br^−^ and H_2_O–Zn^2+^ are 51.5%, 11.0% and 37.4%, respectively (Fig. S22), indicating that both Zn^2+^ and Br^−^ participate in restructuring the hydrogen-bond network away from water–water associations.

**Figure 5.**
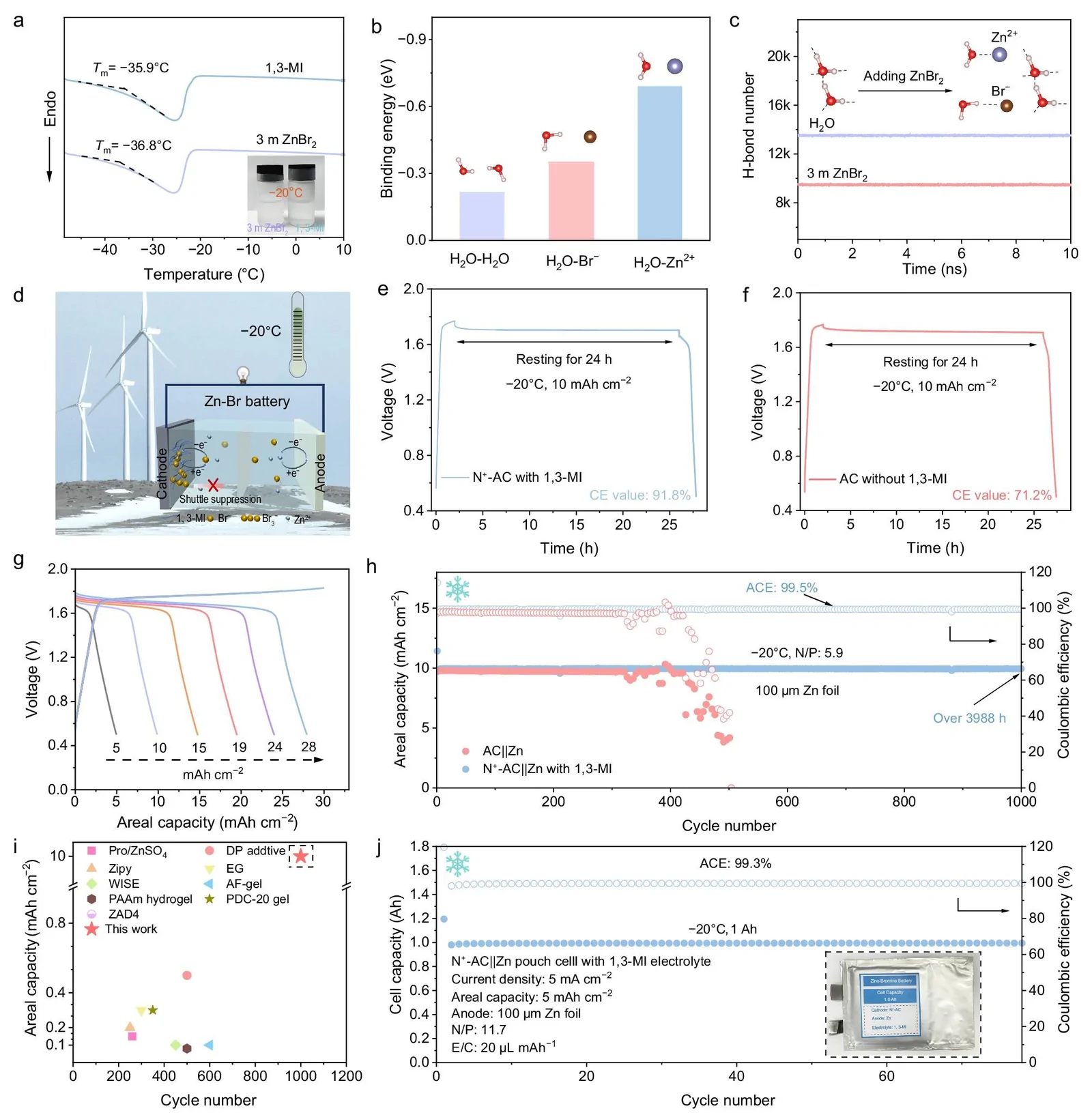
Characterization and electrochemical performance measurements at −20°C. (a) DSC tests of 3 m ZnBr_2_ and 1,3-MI. (b) Binding energy calculations. (c) H-bond counts between H_2_O–H_2_O in pure water and 3 m ZnBr_2_ electrolytes. (d) Images of ZBBs for low-temperature energy storage applications. Self-discharge time–voltage profiles of (e) AC||Zn cell and (f) N^+^-AC||Zn cell with 1,3-MI. (g) Charge–discharge curves of N+-AC||Zn cell with 1,3-MI at various areal capacities. (h) Cycling performance of AC||Zn and N^+^-AC||Zn cells with 1,3-MI at 10 mAh cm^−2^ and 5 mA cm^−2^. (i) Comparison with previous studies on aqueous Zn-based battery systems at −20°C. (j) Cycling test of N^+^-AC||Zn pouch cell with 1,3-MI at 5 mA cm^−2^.

Encouraged by the strong anti-freezing capability and the absence of pronounced ionic conductivity loss at −20°C (Table S4), electrochemical tests were also conducted under subzero conditions (Fig. 5d). Low-temperature self-discharge tests demonstrate that the N^+^-AC||Zn cell with 1,3-MI exhibits superior performance compared to the control AC||Zn cell. After a 24 h rest at −20°C, the N^+^-AC||Zn cell with 1,3-MI retains a high CE of 91.8% (Fig. 5e), markedly outperforming the AC||Zn battery (71.2%, Fig. 5f), and even exceeding its room-temperature CE. Subsequent rate performance tests further reveal that the N^+^-AC||Zn cell with 1,3-MI maintains a high CE of 98.4% at 2 mA cm^−2^, outperforming that of the control AC||Zn cell (93.9%). Upon increasing the current density from 2 to 20 mA cm^−2^, the CE remains highly stable with fluctuations of only 0.6%, reflecting excellent rate stability at low temperature (Fig. S23). Long-term cycling tests indicate that the N^+^-AC||Zn cell with 1,3-MI maintains stable operation for over 3988 h with a high areal capacity of 10 mAh cm^−2^ (Fig. 5h), accompanied by nearly overlapping voltage-capacity profiles across cycles (Fig. S24), evidencing a highly stable reaction interface even at subzero temperatures. In contrast, the AC||Zn cell suffers from rapid capacity fading and fails after 500 cycles. As compiled in Fig. 5i and Table S5, the N^+^-AC||Zn cell with 1,3-MI outperforms most state-of-the-art aqueous Zn-based battery systems at −20°C with respect to areal capacity [41–55]. Moreover, even at a low current density of 1 mA cm^−2^ and an areal capacity of 10 mAh cm^−2^, the N^+^-AC||Zn cell with 1,3-MI maintains robust operation for over 780 h (Fig. S25). Notably, it delivers an ultrahigh areal discharge capacity of up to 28 mAh cm^−2^ at −20°C and sustains durable cycling for >70 cycles (Fig. 5g and Fig. S26). Encouragingly, 1 Ah-scale pouch cells incorporating this strategy also demonstrate stable cycling at −20°C, representing one of the highest reported cell-level capacities for aqueous Zn-Br_2_ batteries under low-temperature conditions (Fig. 5j).

### Enhanced suppression of polybromide shuttling at −20°C

Interestingly, the N^+^-AC||Zn (with 1,3-MI) cell exhibits improved cycling stability and higher CE under subzero temperatures than at room temperature, motivating a closer examination of the temperature dependence of polybromide shuttling. First, *in situ* electrochemical optical microscopy measurements were employed. As shown in Fig. 6a, with a Pt wire cathode, Zn anode and 3 m ZnBr_2_ electrolyte at room temperature, the region surrounding the Pt wire gradually turned yellow during charging. Notably, the yellow zone at room temperature was lighter in color yet spread over a broader area than that observed at −20°C (Fig. 6b), providing direct visual evidence that low temperature markedly suppresses polybromide diffusion. This trend is consistent with Br_3_^−^ diffusion experiments conducted in an H-type electrolytic cell and the temperature-dependent viscosity change of the electrolyte (Fig. S27 and Table S6). In contrast, in the presence of 1,3-MI at −20°C, a distinct oily complex forms and adheres to the Pt-wire surface during charging, effectively blocking polybromide transport from the interphase into the bulk electrolyte (Fig. 6c). The corresponding enhanced discharge time in time–voltage profiles further corroborates this observation (Fig. 6d). To clarify the difference in the complexation ability of 1,3-MI toward polybromides between room and low temperatures, UV-vis spectroscopy was used to compare the solubility characteristics of 1,3-MIBr_3_ at room temperature and at −20°C. After introducing 0.45 wt% 1,3-MIBr_3_ into 3 m ZnBr_2_ electrolyte, Br_3_^−^ absorption intensity in the supernatant is higher at room temperature than at −20°C (Fig. 6e), directly indicating reduced solubility of 1,3-MIBr_3_ and thus enhanced effective complexation/precipitation at low temperature. MD simulations further support the above interpretation. Compared with room temperature (Fig. 6f and Fig. S28a), Br_3_^−^ exhibits more pronounced aggregation at −20°C (Fig. 6g and Fig. S28b), along with markedly suppressed diffusion kinetics (Fig. 6h and Fig. S28c).

**Figure 6.**
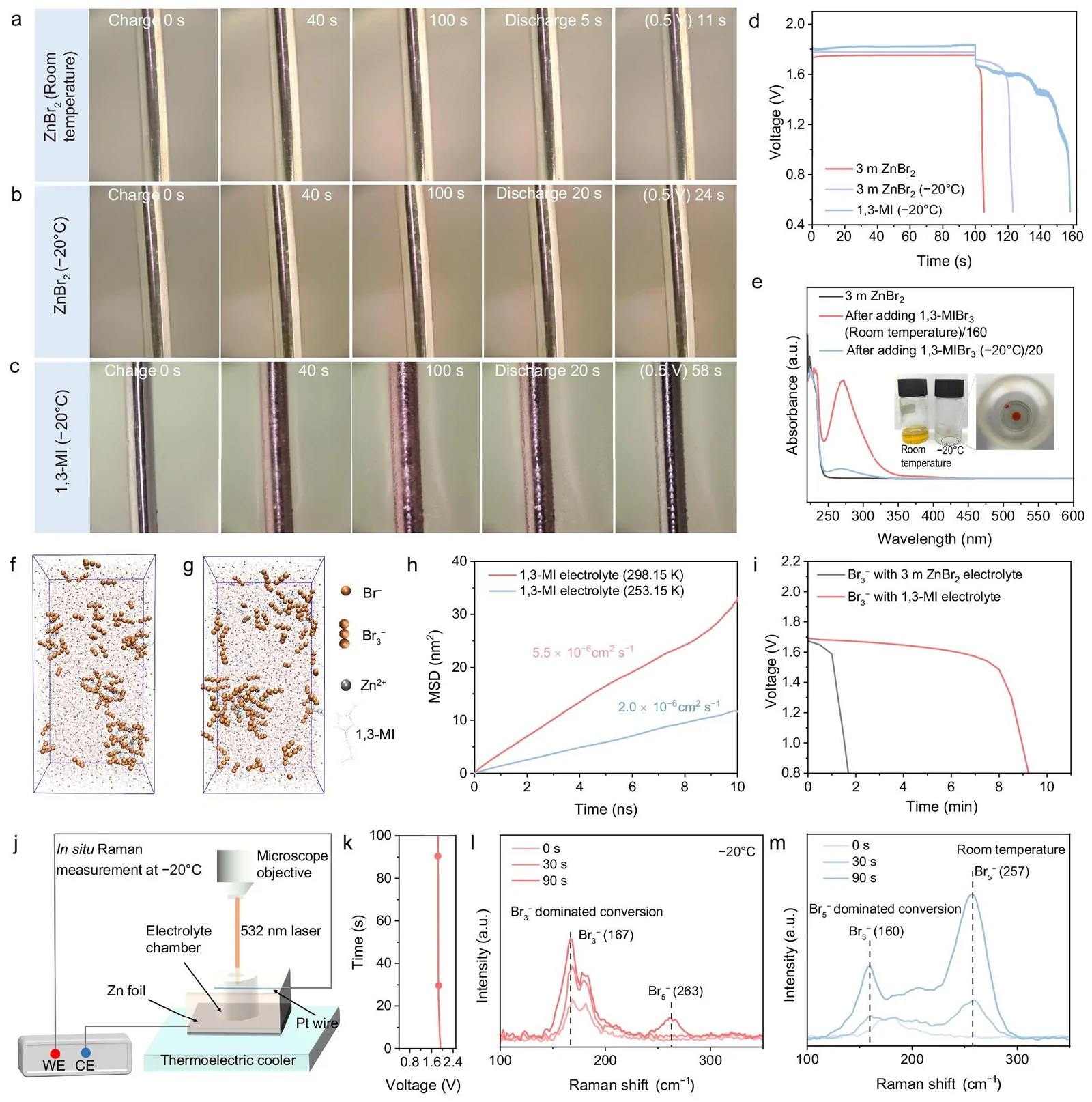
Mechanistic characterization of enhanced polybromide shuttle inhibition at −20°C. *In situ* optical microscopy observation of polybromide evolution around Pt electrode in 3 m ZnBr_2_ electrolyte during charge–discharge at (a) room temperature and (b) −20°C, and in (c) 1,3-MI electrolyte at −20°C, along with (d) corresponding time–voltage curves. (e) UV-vis spectra of 1,3-MIBr_3_ solution at different temperatures (the inset is its optical picture). MD simulations of 1,3-MI electrolyte containing Br_3_^−^ anions at (f) room temperature and (g) −20°C. (h) Mean square displacement of Br_3_^−^ anions at different temperatures. (i) Discharge curves of Br_3_^−^ at −20°C. (j) Device diagram of low-temperature *in situ* Raman measurement. *In situ* Raman spectra of bromine species near the Pt wire electrode in 1,3-MI electrolyte at (l) −20°C and (m) room temperature during charging, along with (k) the corresponding charging curve at −20°C. MSD, mean square displacement.

To investigate the interphase reutilization enhancement effect of Br_3_^−^ by 1,3-MI additive at low temperature, a Br_3_^−^ discharge experiment was designed by using non-porous conductive carbon cloth (CC) as the current collector and 10 mM Br_3_^−^ + 3 m ZnBr_2_ as the blank active material/electrolyte (Fig. 6i). It can be seen that the CC electrode with the 1,3-MI additive achieves a substantially longer discharge time (9.2 min) than that in the blank electrolyte (1.6 min). The results indicate that the dynamic migration and accumulation of 1,3-MI at cathode interphase will generate an ionic electric field (*E*_1,3-MI_), facilitating the recovery and conversion of interfacial polybromide species (Fig. S29). More detailed conversion behaviors of polybromide species promoted by the 1,3-MI additive at −20°C are assessed by *in situ* Raman spectroscopy measurements of Pt wire electrode using a home-made low-temperature electrochemical cell (Fig. 6j). The results indicate that the polybromide species generated are dominated by Br_3_^−^ (167 cm^−1^) at −20°C (Fig. 6k and l), whereas higher-order Br_5_^−^ (257 cm^−1^) are predominant at room temperature (Fig. 6m), demonstrating that low-temperature conditions can enhance the anchoring ability of 1,3-MI toward polybromides, which not only reduces the solubility of polybromide but also effectively inhibits the formation of higher-order polybromides (e.g. Br_5_^−^), and facilitates a unique polybromide transformation mechanism, ultimately ensuring the excellent electrochemical performance of the battery at low temperatures.

## CONCLUSION

In summary, a bulk-to-interphase synergistic regulation strategy is rationally designed to precisely suppress polybromide shuttling during both charge and discharge. In the cathode bulk, an N^+^-AC host rich in cationic sites strengthens micropore electrostatic confinement of polybromide anions, thereby inhibiting their leaching from the cathode interior to the cathode/electrolyte interface during charging. At the interphase, the 1,3-MI additive not only anchors polybromides at the interface to impede diffusion into the bulk electrolyte, but also accumulates at the interface during discharge to form an ionic electric field (*E*_1,3-MI_) opposite to the electrode electric field (*E*_electrode_). This electric-field-responsive 1,3-MI accumulation layer promotes the recovery and reduction of polybromide anions in the interphase, mitigating anode-directed electromigration. Theoretical calculations and *in situ* characterizations collectively substantiate the effectiveness of this field-aware, spatially resolved design. Notably, at −20°C, the strengthened complexation of 1,3-MI with polybromides together with the intrinsically slowed polybromide diffusion kinetics further enhances shuttle suppression. Consequently, the anode-free N^+^-AC||Ti cell with 1,3-MI sustains stable cycling for over 2000 cycles at 10 mAh cm^−2^. In addition, the N^+^-AC||Zn cell with 1,3-MI can deliver an ultrahigh discharge areal capacity of 28 mAh cm^−2^ at −20°C and exhibits negligible capacity decay over 1000 cycles at 10 mAh cm^−2^. Impressively, an Ah-level N^+^-AC||Zn pouch cell with 1,3-MI operates stably at both room temperature and −20°C, underscoring the practical feasibility of this strategy under complex climatic conditions. More broadly, this bulk-to-interphase, electric-field-aware regulation concept may be extendable to other static halide-based chemistries limited by active-species shuttling, offering a general pathway toward high-performance, low-cost energy-storage systems for grid-scale deployment.

## Supplementary Material

nwag393_Supplemental_File
