# Supplementary material for: Bulk-to-interphase polybromide confinement and electric-field-responsive recovery enable Ah-level aqueous zinc-bromine batteries at room/low temperatures
Source: Natl Sci Rev. 2026 Jun 25;13(18):nwag393. doi: 10.1093/nsr/nwag393 (PMC13622346; doi:10.1093/nsr/nwag393)
Supplement: Supplementary file 1 [file nwag393_supplemental_file.pdf]

Supporting Information

Bulk-to-interphase polybromide confinement and electric-field-responsive recovery enable Ah-level aqueous Zinc-bromine batteries at room/low temperatures

Qilong Wu, Peiyao Wang, Hong Lin, Fanxiang Meng, Chenxi Sun, Jinbao Zhao and Yang Yang*

State Key Laboratory of Physical Chemistry of Solid Surfaces, State-Province Joint Engineering Laboratory of Power Source Technology for New Energy Vehicle, College of Chemistry and Chemical Engineering, Xiamen University, Xiamen 361005, China

***Corresponding author.** E-mail: yangyang419@xmu.edu.cn

Table of contents

Section 1. Experimental Section 1-6

Section 2. Supplementary Figures 7-36

Section 3. Supplementary Tables... 36-41

Section 4. Supplementary References.. 41-45

Section 1. Experimental Section

Materials

All reagents and starting materials were used as received without any further purification. $ZnBr_2$ (Macklin, 98%); 1-Butyl-3-methylimidazolium chloride (Macklin, 90% aqueous solution); Actived carbon (AC); Poly(diallyldimethylammonium chloride) were purchased from Tianjin Hiens Biochemical Technology Co. The Polytetrafluoroethylene (PTFE) used in this work is a commercial product (POLYFLON PTFE F-104, DAIKIN INDUSTRIES, LTD.). The Br_3^- solution is prepared as reported [1]. The resulted 120 mM Br_3^- solution is in dark orange. And 1, 3-$MIBr_3$ was obtained by mixing a 90% 1, 3-MI solution with a Br_3^- solution, followed by product isolation.

Preparation of N^+-AC

Poly(diallyldimethylammonium chloride) (N^+) with varying masses (0.025 g, 0.05 g, 0.1 g, and 1.0 g) was dissolved in 100 mL of deionized water, followed by thorough stirring to form a homogeneous solution. Subsequently, activated carbon (AC) was added into the above solution, and the mixture was continuously stirred for 2 h. After that, the resultant mixture was subjected to centrifugation, rinsed thoroughly with deionized water and ethanol, and finally dried overnight at 60 °C to obtain N^+-AC.

Preparation of AC and N^+-AC self-standing electrode

First, N^+-AC (7 g) and the conductive agent super P (2 g) were mixed to form a homogeneous powder. Subsequently, 0.6 g of PTFE was added to the as-prepared powder, followed by pre-fibrillation under shear force to enhance the binding between N^+-AC and the conductive agent. The mixture was then compressed and repeatedly rolled 2-3 times using a double-roll calender until a thickness of 1.5 mm was achieved, yielding a free-standing N^+-AC electrode sheet. And the electrode was cut into 12-mm-diameter circular discs to assemble Zn-Br_2 full cells. AC electrodes were prepared in the same process using AC powder.

Characterizations

Zeta potential measurements were obtained from the Zetasizer instrument (90 Plus PALS, Brookhaven Instrument). Field scanning electron microscope (FESEM, Zeiss Merlin Gemini) was used to analyze X-ray energy spectrum (EDS) of the sample. The in-situ optical images were acquired on CX40M metallographic microscope (NINGBO SUNNY INSTRUMENTS). Differential scanning calorimetry (DSC) was conducted by Mettler-Toledo DSC3. Cyclic Voltammetry (CV) tests were conducted by using the electrochemical workstation (CHI 660D). The electrochemical impedance spectroscopy (EIS) was measured by Solartron electrochemical workstation. The electrochemical workstation (CHI440B) was coupled with an electrochemical quartz crystal microbalance (EQCM). The ^{1}H nuclear magnetic resonance (NMR) measurements were conducted on a 500 MHz NMR spectrometer (Bruker AVANCE NEO 500).

In the EQCM measurement, QSense Explorer (Biolin Scientific) and the electrochemistry module (QEM 401) were employed to obtain the EQCM-D results. Gold-coated AT-cut quartz crystals (5 MHz fundamental frequency, active sensing area of 0.785 cm^{-2}, QSX 301 Au) were employed to track the mass and dissipation factor changes of the working electrode. To fabricate the Au@N^{+}-AC quartz crystal electrodes, a N^{+}-AC slurry containing N^{+}-AC and PVDF (mass ratio 9:1) was drop-cast onto Au sensor surface. The 2 wt% 1, 3-MI aqueous solution (no $ZnBr_2$) and a Pt electrode were used as the electrolyte and counter electrode, respectively. The electrochemical workstation (CHI 440B) was used to realize cyclic voltammetry control and electrochemical data acquisition. The potential was first swept anodically from the open-circuit potential (0.22 V vs. Ag/AgCl) to 0.5 V at a scan rate of 0.1 mV s^{-1}, followed by a cathodic sweep to -0.15 V. The EQCM measurements in pure water were conducted under conditions similar to those for the 1, 3-MI solution. The potential was first scanned anodically to 0.5 V and then cathodically to 0.1 V to avoid hydrogen evolution. According to the Sauerbrey equation, the mass change was calculated as: $\Delta m=-C\times\Delta f/n$. The C is the sensitivity constant, which is 17.7 ng Hz^{-1} cm^{-2} here. The n is the odd multiple of frequency, thus the n=1 for base frequency and n=3 for triple frequency (f_3). In this work, the f_3 is employed as the excitation frequency, thus the 1 Hz increase in frequency corresponded to a mass decrease of 4.63 ng.

All Raman spectroscopy measurements were used Horiba LabRAM HR Evolution (France) with a laser wavelength of 532 nm. The in situ Raman spectroscopy of AC||Zn, N^+-AC||Zn and N^+-AC||Zn cells with 1, 3-MI was performed using a home-made Raman cell. And the batteries were charged to10 mAh cm^{-2} at 20 mA cm^{-2}. For in situ Raman spectroscopy measurements at room temperature and -20ºC with a Pt wire as the cathode, the measured region is the electrolyte near the Pt wire, and the cell was operated at a constant current density of 0.33 mA cm^{-1}.

All UV-vis spectroscopy measurements were used UV-2600 (Japan). For in situ UV-vis measurements of AC||Zn, N^+-AC||Zn and N^+-AC||Zn cells with 1, 3-MI, titanium mesh as the current collector. And the electrode featured a thickness of 500 μm and an area of 2 cm^2, and was charged to 1.5 mAh cm^{-2} and then discharged to 0.5 V.

Electrochemical measurements

Swagelok® type cells were assembled in the air for electrochemical tests, glass fiber filter (GF-D, Whatman) as the separator, 3 m $ZnBr_2$ or 3 m $ZnBr_2$+2 wt% 1-butyl-3-methylimidazolium chloride aqueous solution (1, 3-MI) as the electrolyte. The cathode was soaked in the electrolyte prior to use and the total theoretical capacity contributed by the $ZnBr_2$ electrolyte in the full cell is approximately 43 mAh cm^{-2}, including ~16 mAh cm^{-2} from the infused electrolyte and ~27 mAh cm^{-2} from the electrolyte adsorbed within the electrode. For Zn-Br_2 full cells assembly, 100 μl electrolyte and Zn foil (100 μm unless otherwise noted) were used. And in the case of the anode-free battery configuration, 50 um titanium (Ti) foil served as the anode current collector. The electrochemical tests were performed under constant current conditions, charging to a set capacity and then discharging to 0.5 V. The galvanostatic charge-discharge tests were conducted on the Neware battery system.

In the in situ electrochemical optical microscopy measurements, a 20-μm Pt wire and a 100-μm zinc foil were employed as the cathode and anode, respectively. The cell was subjected to one pre-cycle prior to the test. Galvanostatic charge/discharge was performed in the optical cell with current density of ~16.7 μA mm^{-1}.

Assembly of Pouch Cells

The self-standing electrodes with a thickness of 0.9 mm were fabricated in batches and cut into 10 × 10 cm^2 sheets, which were laminated onto the two sides of titanium

mesh current collectors (10 × 10 cm^2) to obtain the 1.5 mm positive electrodes of pouch cells. The 100 μm zinc foil (10.5 × 10.5 cm^2) was employed as the anode, and its surface was modified with a CuIn alloy layer. The Zn@CuIn electrode was fabricated via a chemical displacement method [2]. Two pieces of Zn@CuIn foils and one double-sided cathode were assembled into a pouch cell, where GF-D glass fiber (11 × 11 cm^2) was used as the separator and 20 ml 1, 3-MI solution (containing 3 m $ZnBr_2$ and 2% 1-butyl-3-methylimidazolium chloride) served as the electrolyte. Finally, the cell was sealed using an aluminum-laminated film as the pouch casing. The assembly of the 3 × 3 cm^2 pouch cell for powering toy cars follows a similar process.

Theoretical calculations

Quantum chemistry (QC) calculation was performed with Gaussian09 E.01 software in this work.[3] The B3LYP-D3[4,5] functional and def2-SVP[6] basis set was used to optimize the geometry of all structures. The vibration analysis was performed at the same theoretical level to ensure that all structures are energy minima. The high-precision single-point energy was calculated by Gaussian09 E.01 at the B3LYP-D3/def2-TZVPP[7] theoretical level. Solvent effects of water (ε = 78.4) was considered by SMD[8]solvation model. The restrained electrostatic potential (RESP)[9] were calculated through Multiwfn[10,11] software and VMD[12] software was used to visualize the systems.

All first-principles calculations based on density functional theory (DFT) were conducted through the Vienna Ab-initio Simulation Package (VASP)[13]. The Perdew Burke-Ernzerhof (PBE) functional within the generalized gradient approximation (GGA) was used to describe the exchange-correlation interaction.[14] Projector augmented wave (PAW) methods are used for pseudopotentials.[15] The DFT-D3 method is used to describe the van der Waals interaction.[16] The VASPKIT code is used for the post-processing of the VASP computational data.[17] The plane-wave basis set cutoff energy was set to 500 eV. All molecular structures were optimized using the Conjugate Gradient method. The total energy convergence criterion for electron relaxation was set to 1.0×10^{-5} eV, and the convergence criterion for ion relaxation was 0.02 eV/Å. The γ-centered Monkhorst-Pack scheme with a K-point resolved value of 0.06 π/Å is used in all DFT computations. The differential charge density was calculated according $\Delta\rho = \rho12 - \rho1 - \rho2$, where ρ1, ρ12 and ρ2 are the charge density

of bromine species, 1, 3-MI with and without bromine species, respectively. The yellow color represents the electron accumulation, and cyan color represents the electron depletion. The isosurface with a unit of 0.001 e bohr-3

Molecular dynamics (MD) simulation was performed through Gromacs2018.8[18] software to study the Zn/electrolyte interface structure at different potentials. The small molecules (1,3-MI , water, $ZnBr_2$ and N^+) were reasonably optimized via Gaussian09 E.01[3] software with a level of B3LYP-D3[4,5] functional and def2-SVP[6] basis set before simulations. The force field parameters of Zn^{2+} ions and SPC/E[19] water model was obtained with Amber99SB[20] force field.The C-layers, 1,3-MI, Br^- and Br_3^- were generated with Sobtop software[21]. RESP atom charges were used to describe electrostatic interactions. The composition of the simulation box for the Electrolyte bulk is 3 M $ZnBr_2$ + 2 wt% 1, 3-MI, including 26 1,3-MI, 405 $ZnBr_2$ and 7500 H_2O. The composition of the simulation box for the cathode interface is 3 M $ZnBr_2$, including 1920 C atoms, 60 3-N^+(Three monomers connected together), 378 $ZnBr_2$ and 7000 H_2O. An 2-layer slab containing 1920 C atoms was built in Material Studio[22,23], which used as the model of [0 0 1] surface of the Active Carbon electrode. A constraint potential (force constant = 1×10^3 kJ mol^{-1} nm^{-2}) in the x, y and z axes for C atoms in the electrode was used to maintain the rigid structure of C-layer. The boxes of $ZnBr_2$ aqueous solution were built by filling molecules randomly. The initial structures were modelled via Packing Optimization for Molecular Dynamics Simulations (Packmol) program[24] and the periodic box was set to 5*5*10 and 5*5*12 nm^3. All the boxes were first submitted to energy minimization by using the 100000-step steepest descent method in order to avoid unreasonable contact of system. The equilibrium simulation was carried out with NPT ensemble at 253.15 K or 298.15 K and 1 bar for 10 ns, using the Berendsen barostat, the non-bonding cutoff radius was 1 nm. The production simulation was carried out with NPT ensemble at 253.15 K or 298.15 K for 10 ns, using the Parrinello-Rahman barostat. The simulation bulk to cathode interphase incorporates an external electric field of -0.01 V/nm along the z-axis. The RDF function, mean square displacement (MSD) and distribution of number density was calculated through Gromacs2018.8. The diffusion coefficients of Br_3^- can be estimated from the slope of the MSD curves by the Einstein relationship and analyzed through the 10 ns simulation. VMD[13] software was used to visualize the systems, obtain the ion association state and count hydrogen bonds (angle between H atom, hydrogen bond donor and acceptor

less than 30 degrees, and distance between donor and acceptor less than 3.5 Å). A time step of 2 fs was used for all simulations.

Section 2. Supplementary Figures

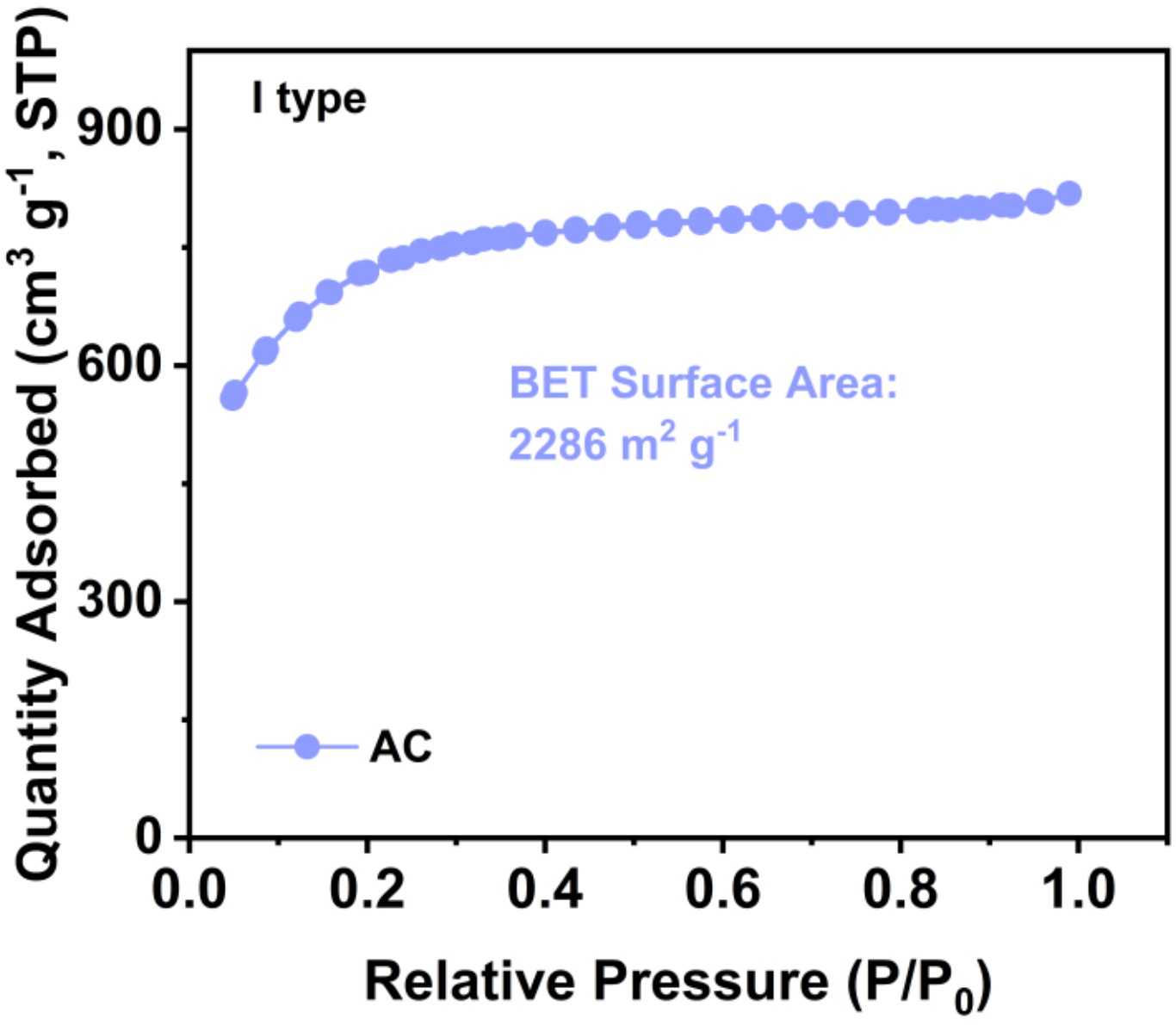

Figure S1. N_2 sorption isotherms curve of AC.

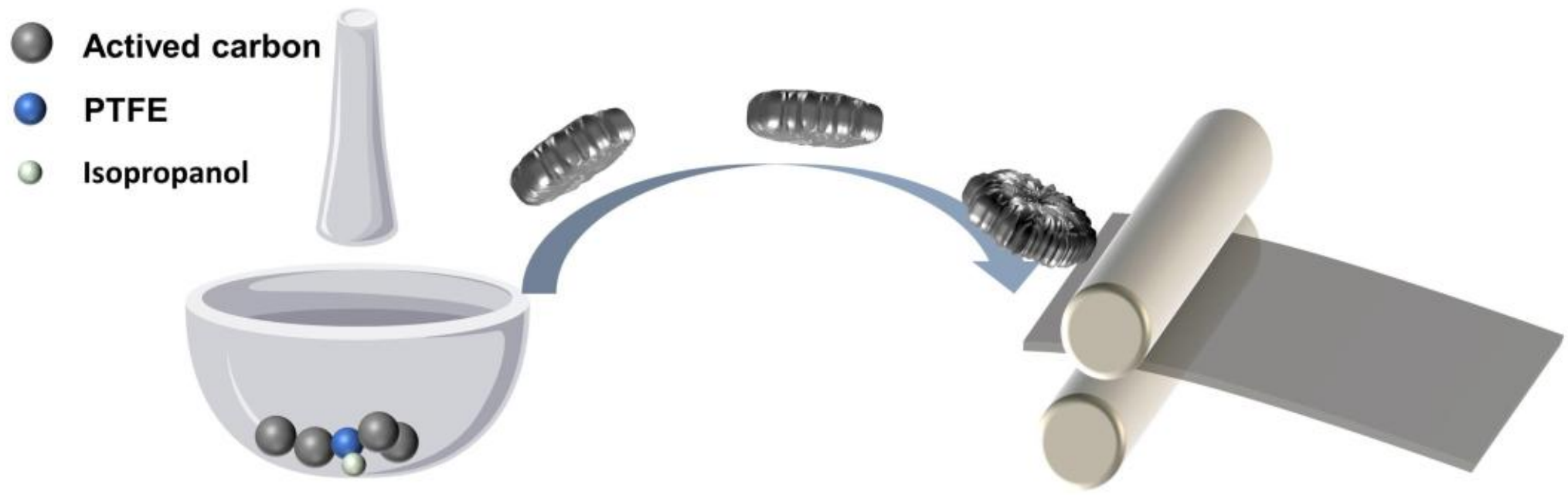

Figure S2. Schematic diagram of dry electrode preparation.

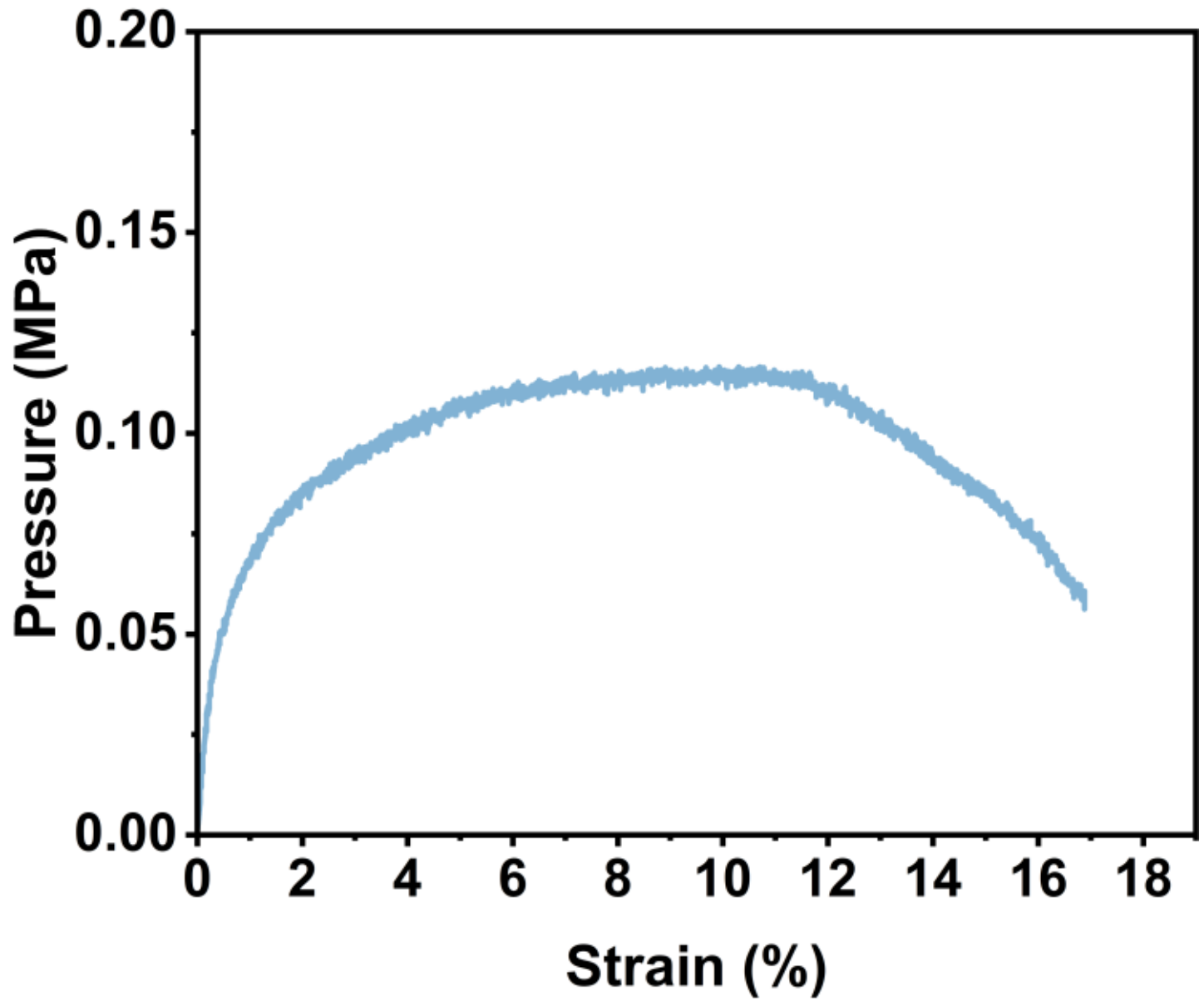

Figure S3. Tensile testing.

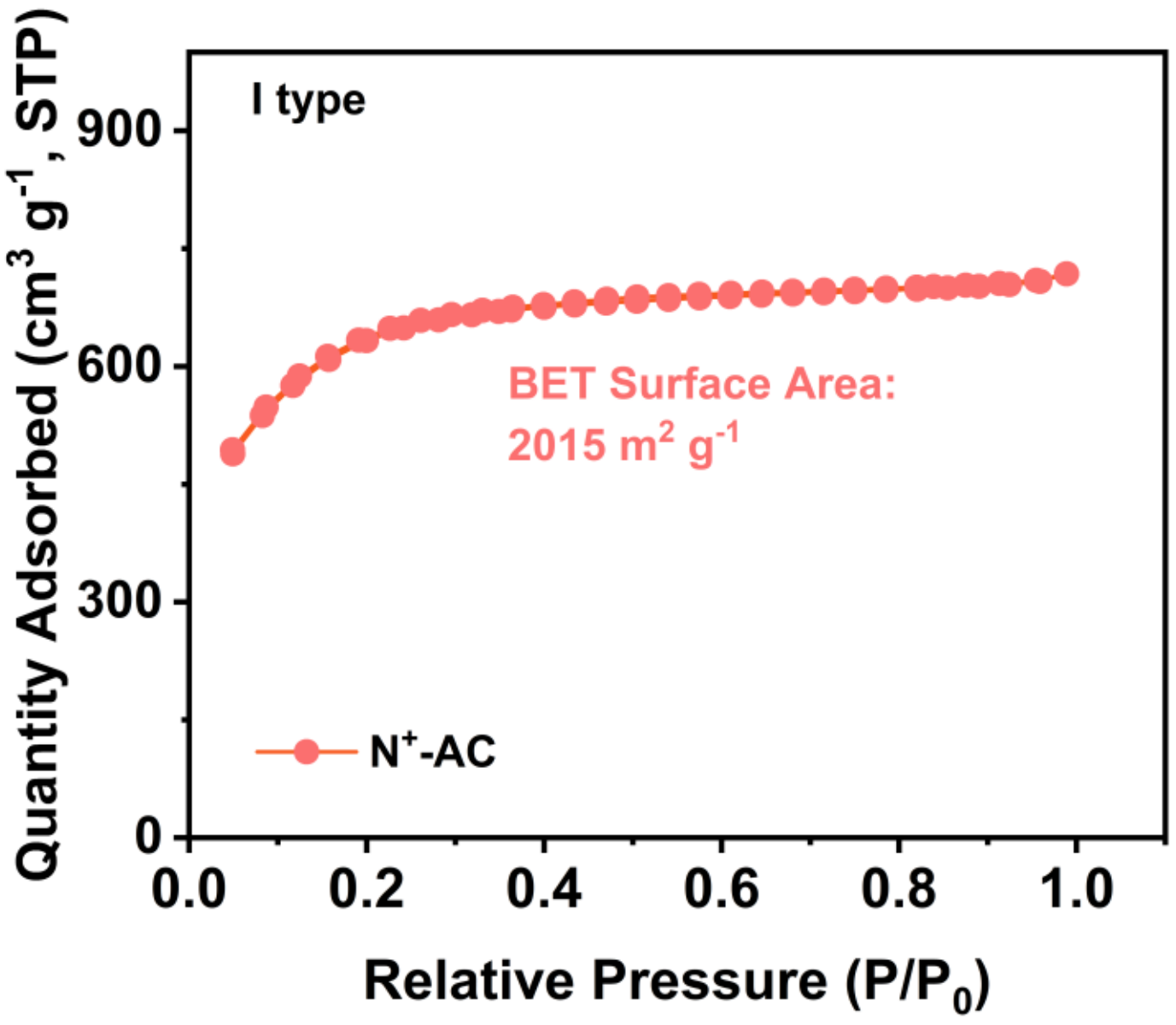

Figure S4. N_2 sorption isotherms curve of N^+-AC.

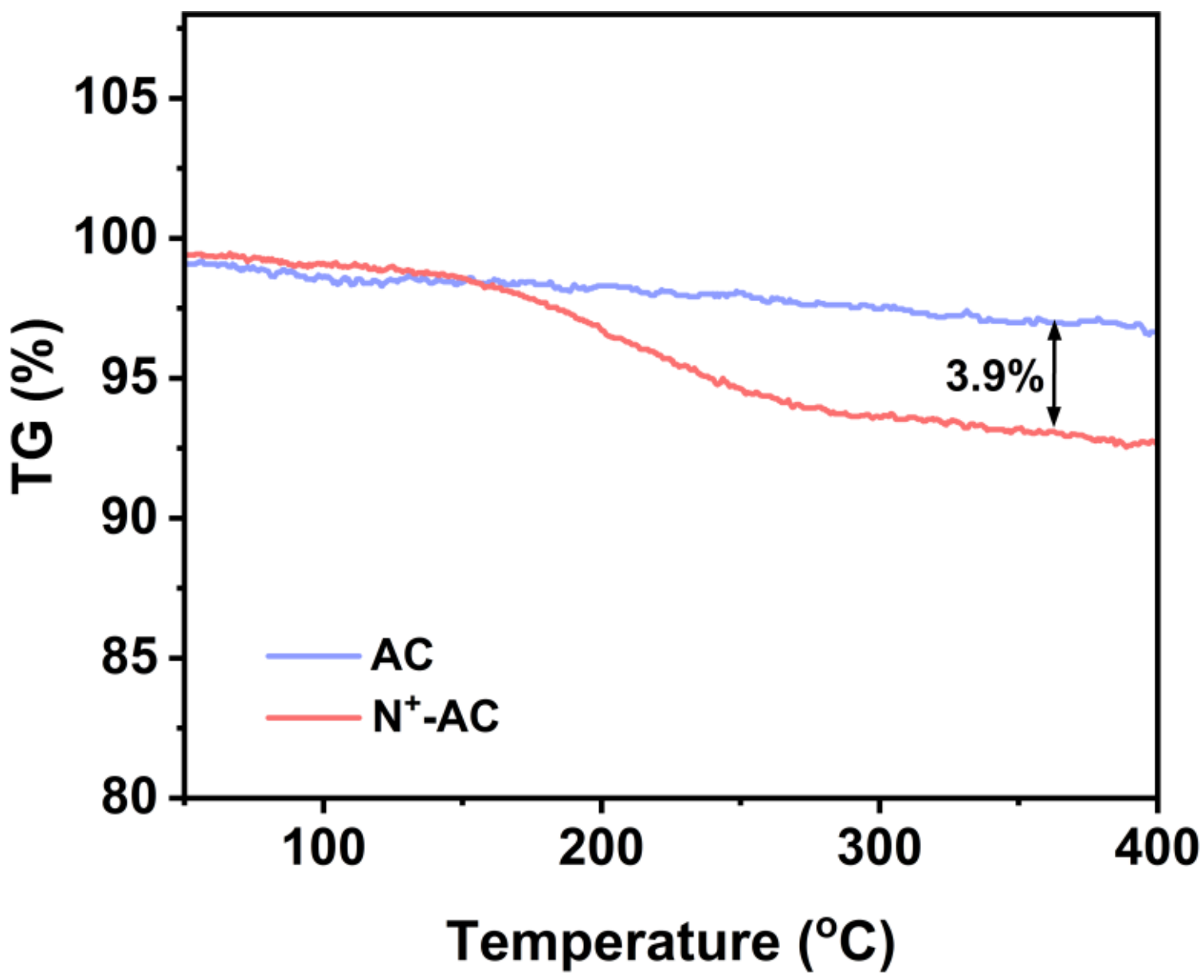

Figure S5. Thermogravimetric (TG) curves of AC and N^+-AC.

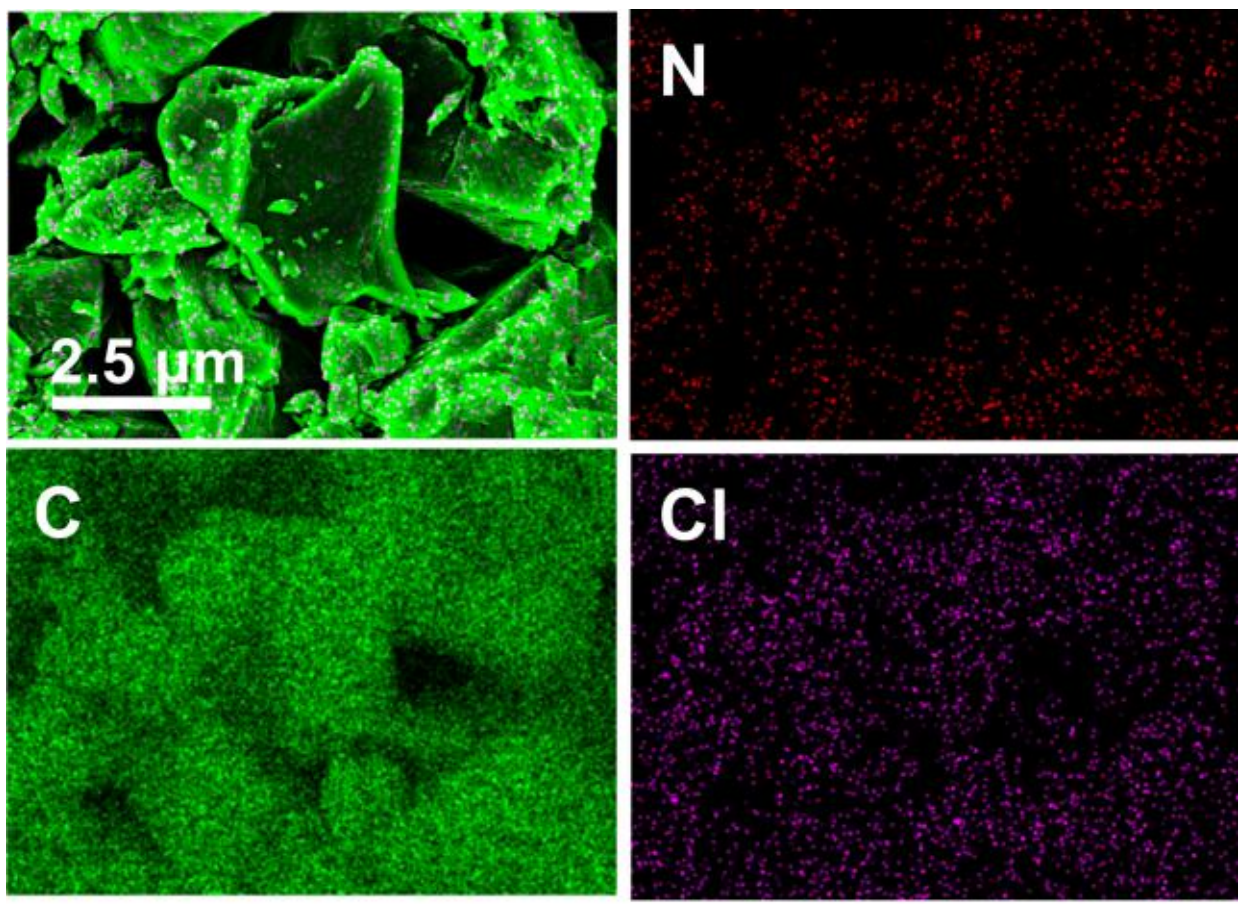

Figure S6. Elemental mapping images of N$^+$-AC.

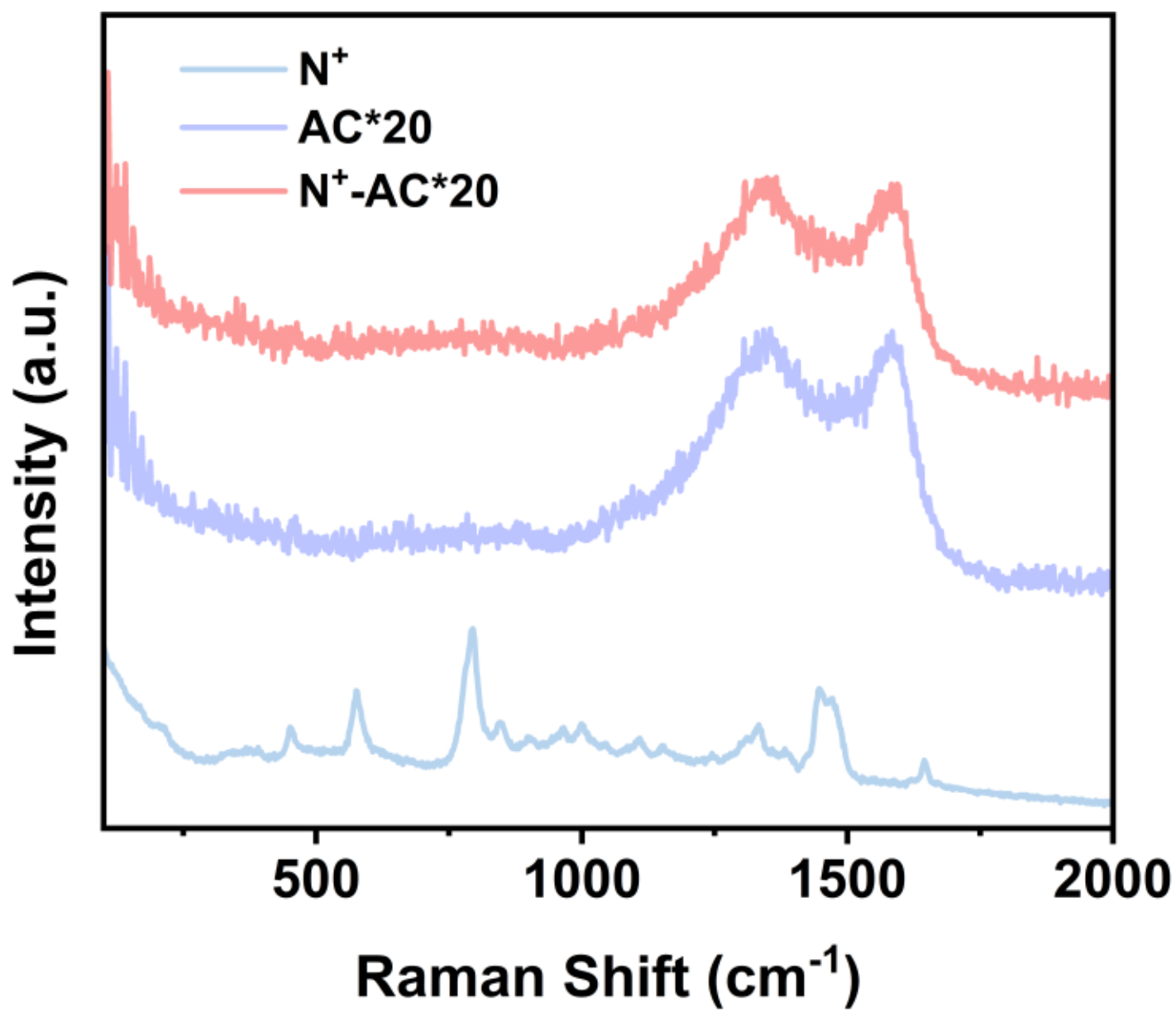

Figure S7. Raman spectra of N^+, AC and N^+-AC powders.

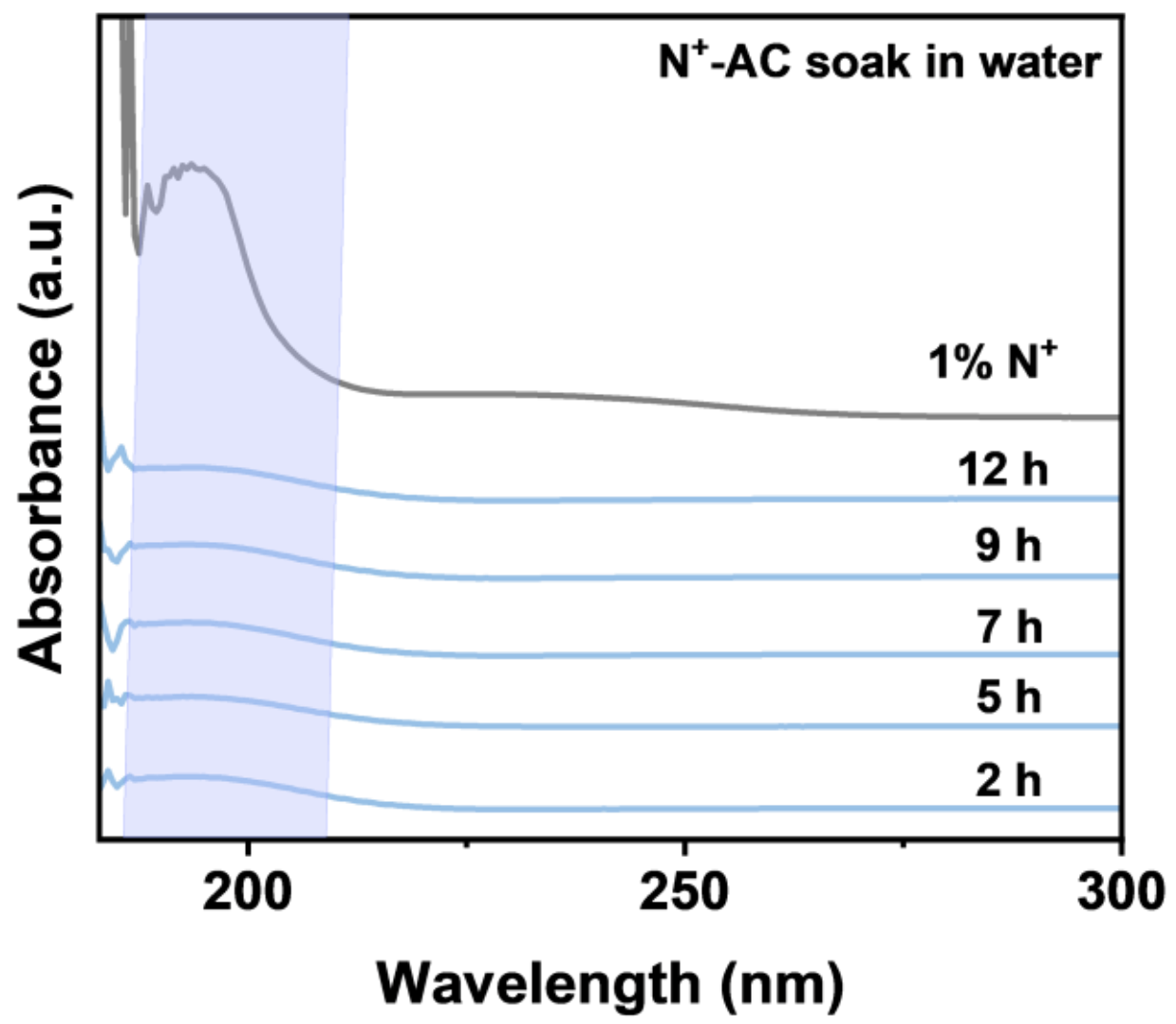

Figure S8. UV-vis spectral variations of N^+-AC with different immersion times in deionized water.

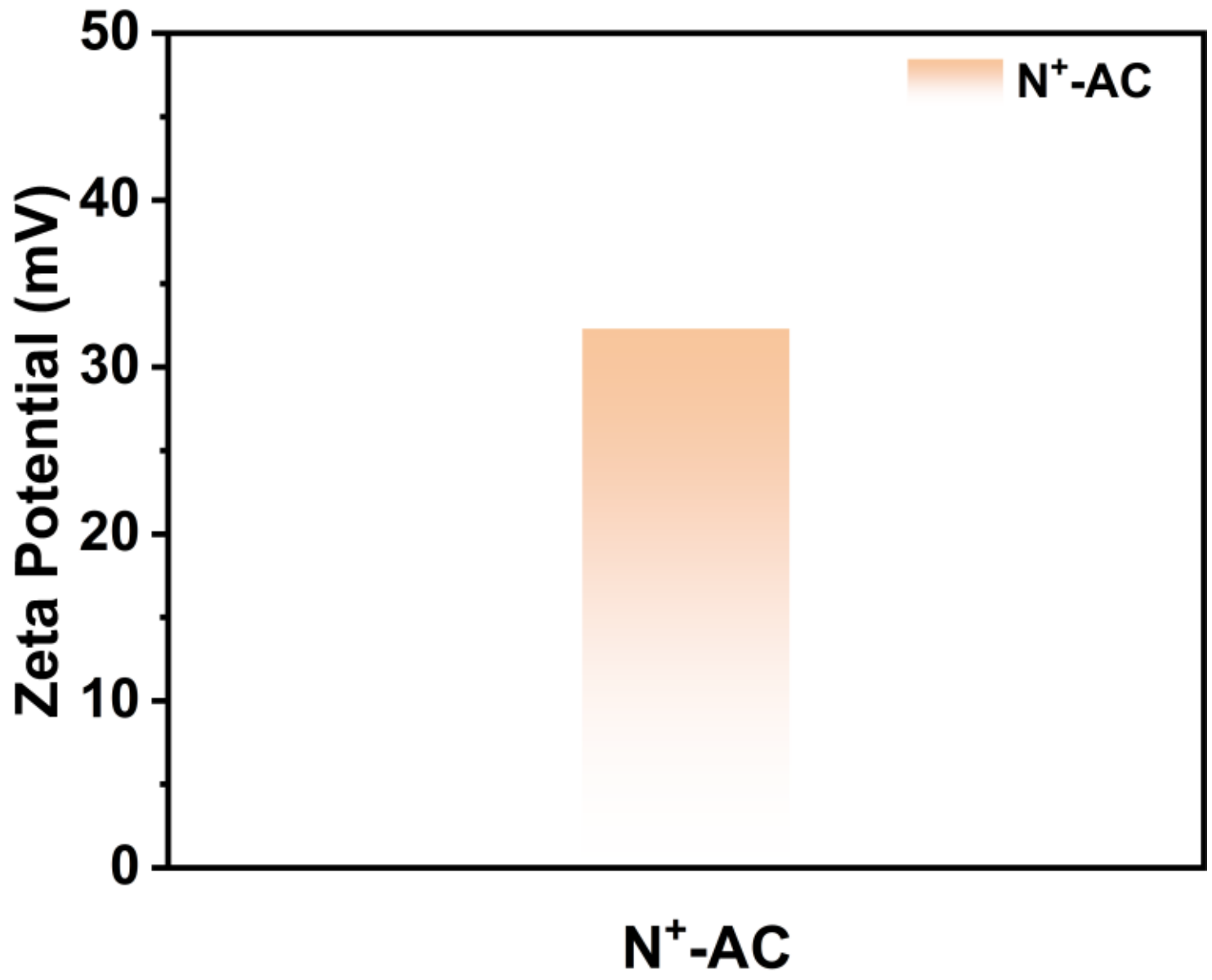

Figure S9. Zeta potential of N^+-AC after 12 h immersion in deionized water.

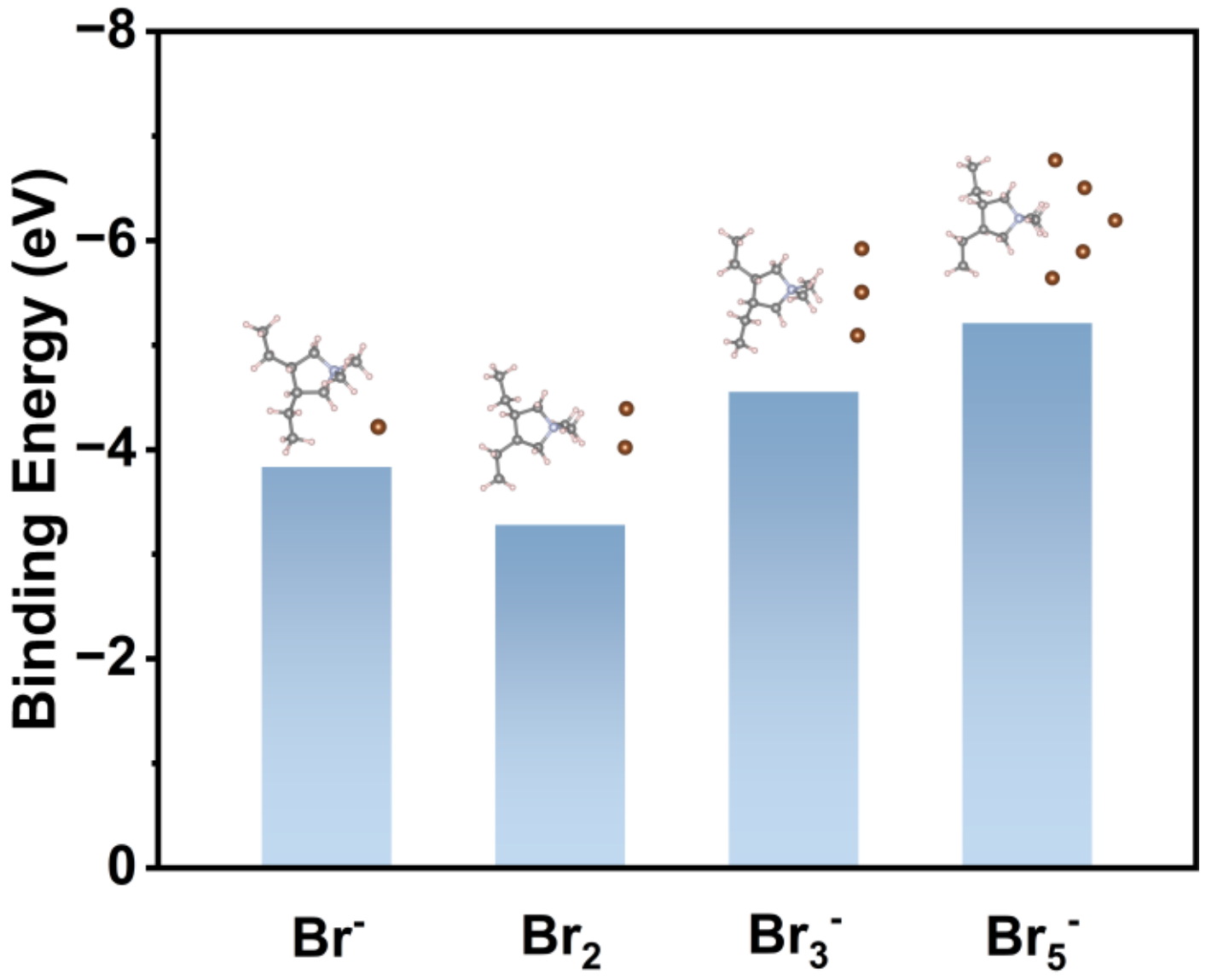

Figure S10. The binding energy calculations of N^+ with bromine species (Br^-, Br_3^-, Br_5^-, Br_2).

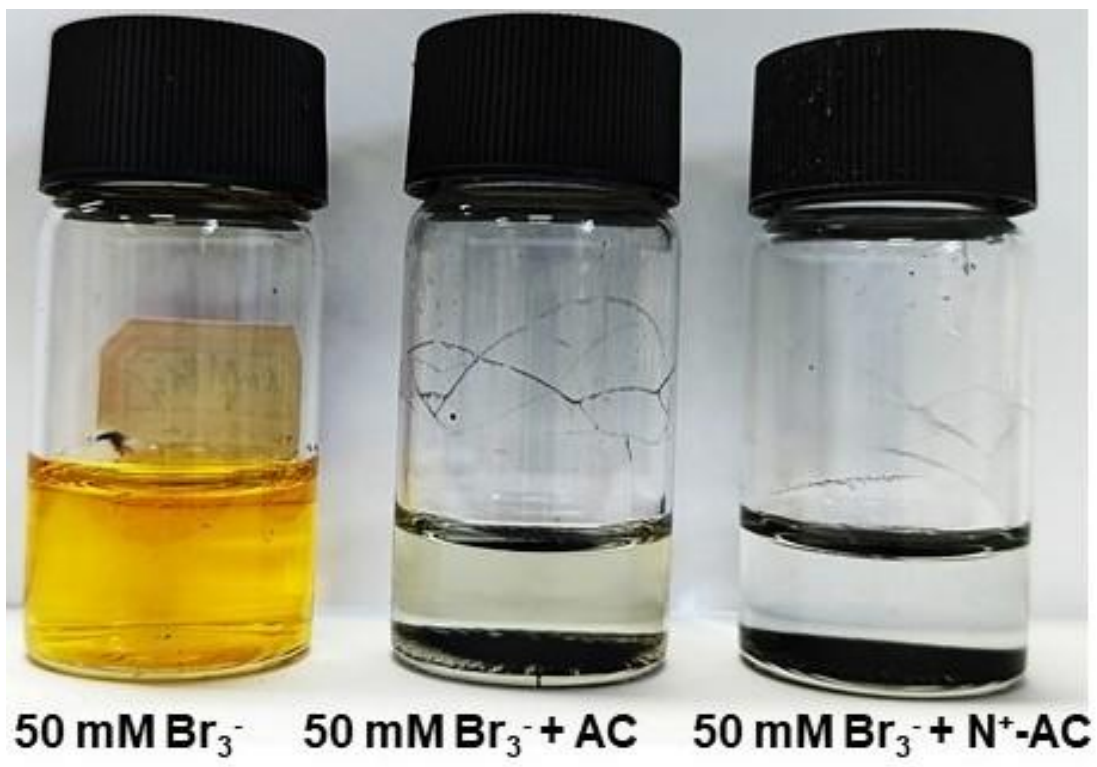

Figure S11. The optical picture of 50 mM Br_3^-, 50 mM Br_3^-+AC and 50 mM Br_3^-+N^+-AC.

Note: Specifically, equal masses of AC and N^+-AC were dispersed in 50 mM solutions, followed by overnight equilibration, to evaluate their polybromide adsorption performance. Notably, the solution treated with N^+-AC became nearly transparent, whereas the AC-treated counterpart retained a distinctly visible yellow coloration. These visual observations directly confirm the enhanced adsorption capacity of N^+-AC toward polybromide species.

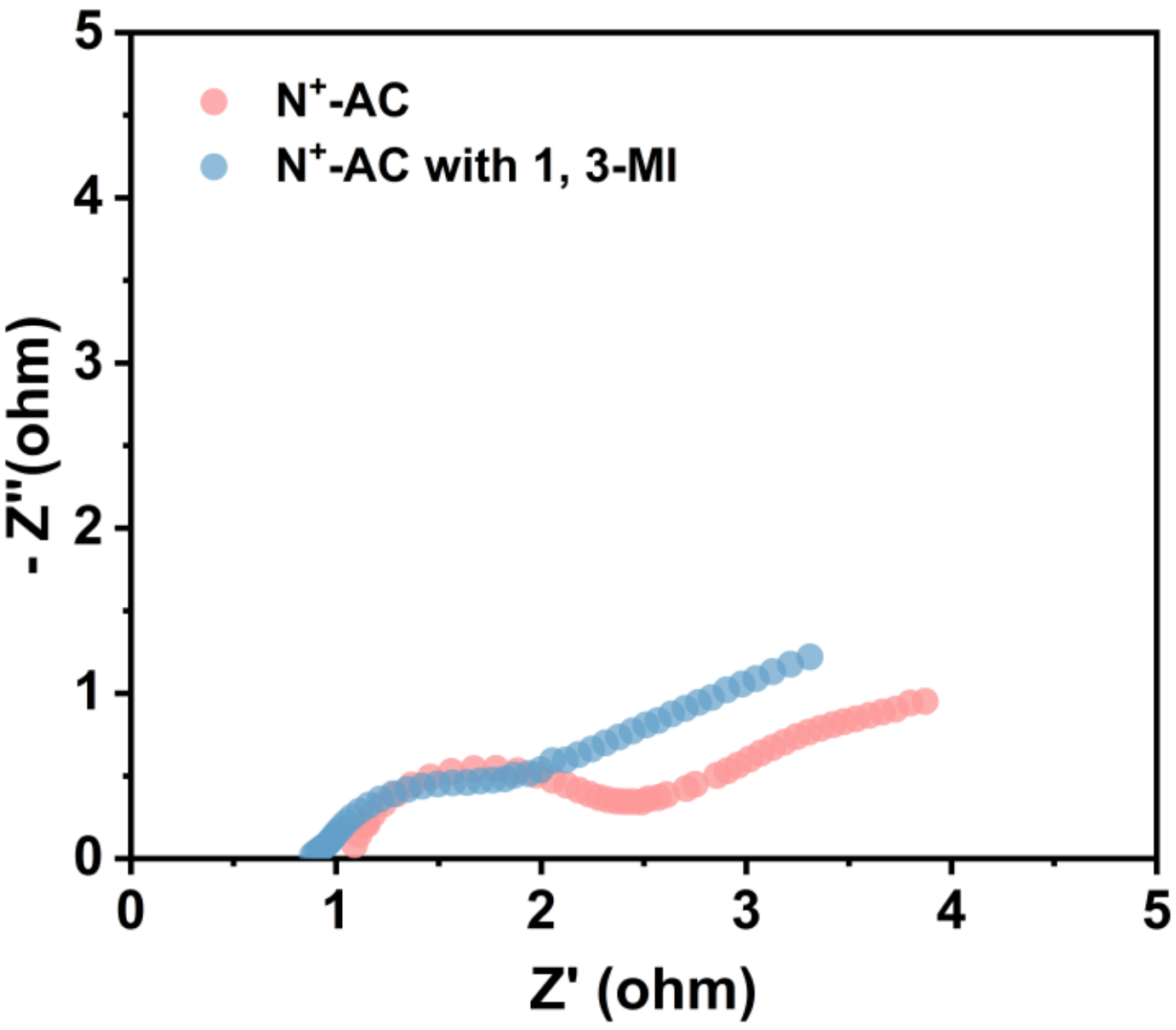

Figure S12. Electrochemical Impedance Spectroscopy (EIS) measurements of N^+-AC||Zn cell and N^+-AC||Zn cell with 1, 3-MI.

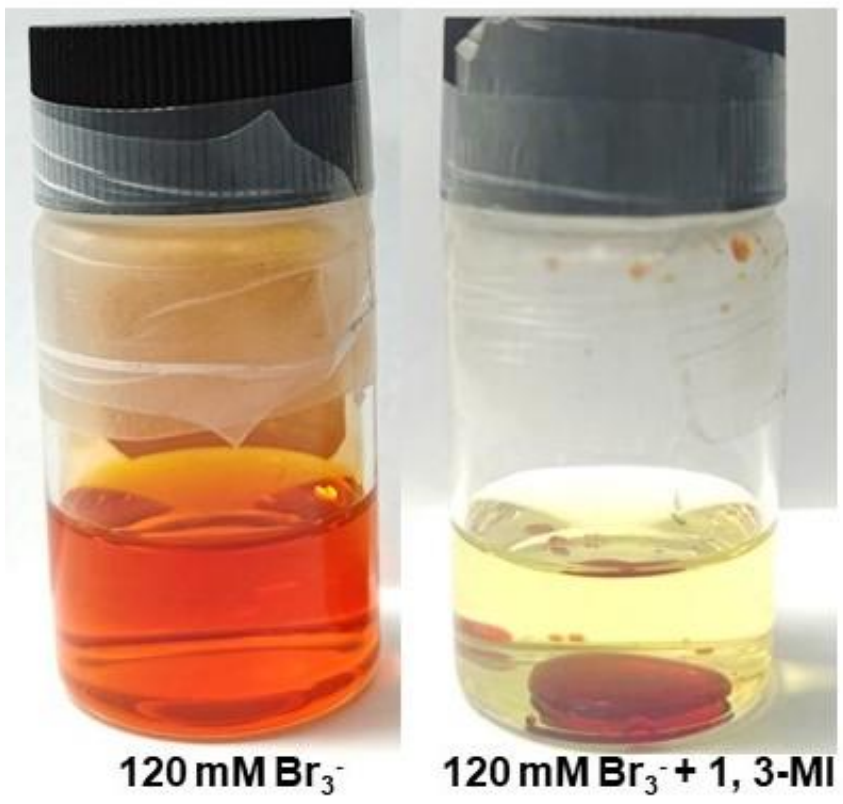

Figure S13. The optical pictures of 120 mM Br_3^- solution and 120 mM Br_3^- solution with 1, 3-MI.

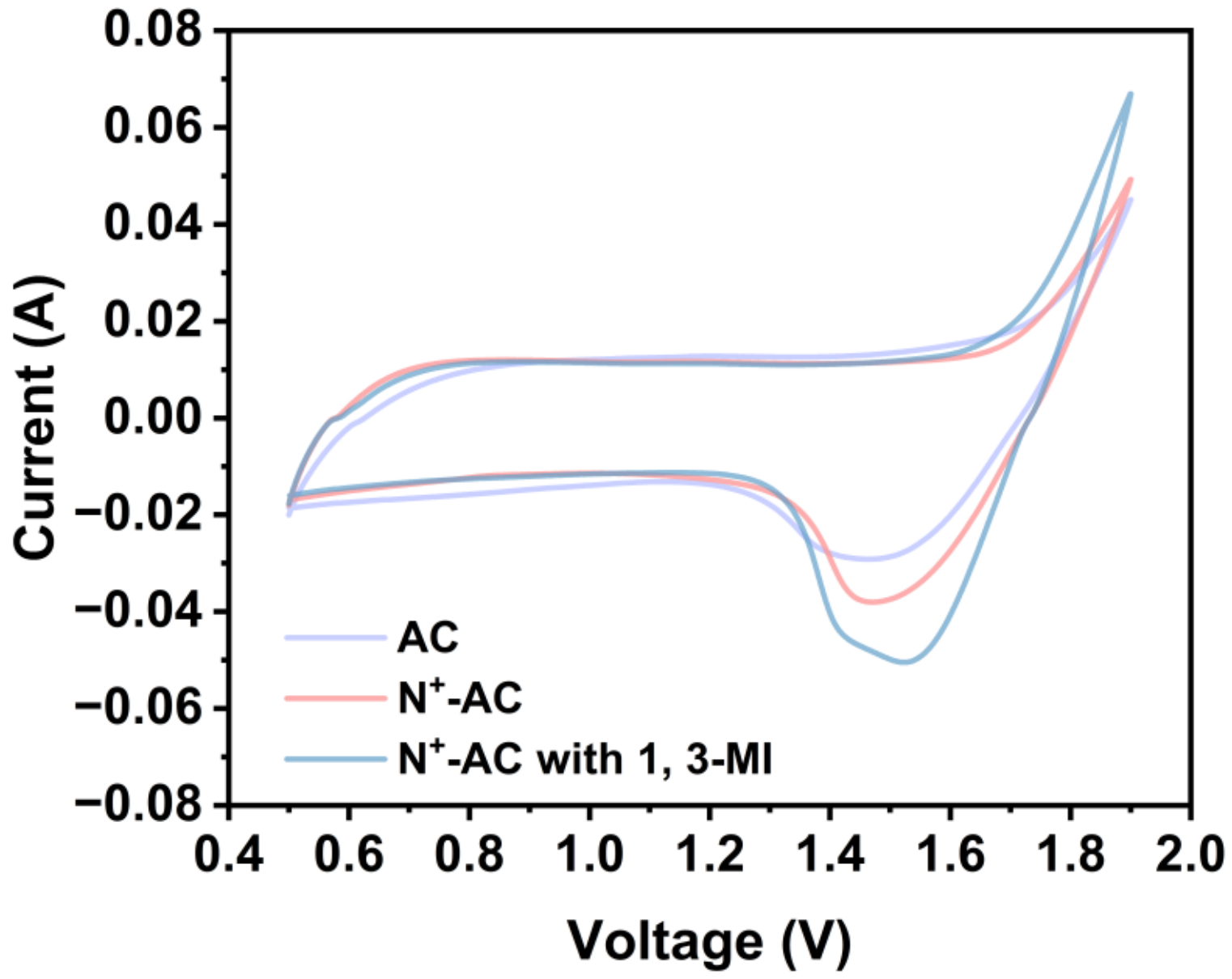

Figure S14. CV measurements of AC||Zn, N^{+}-AC||Zn and N^{+}-AC||Zn cells with 1, 3-MI at 1 mV s^{-1}.

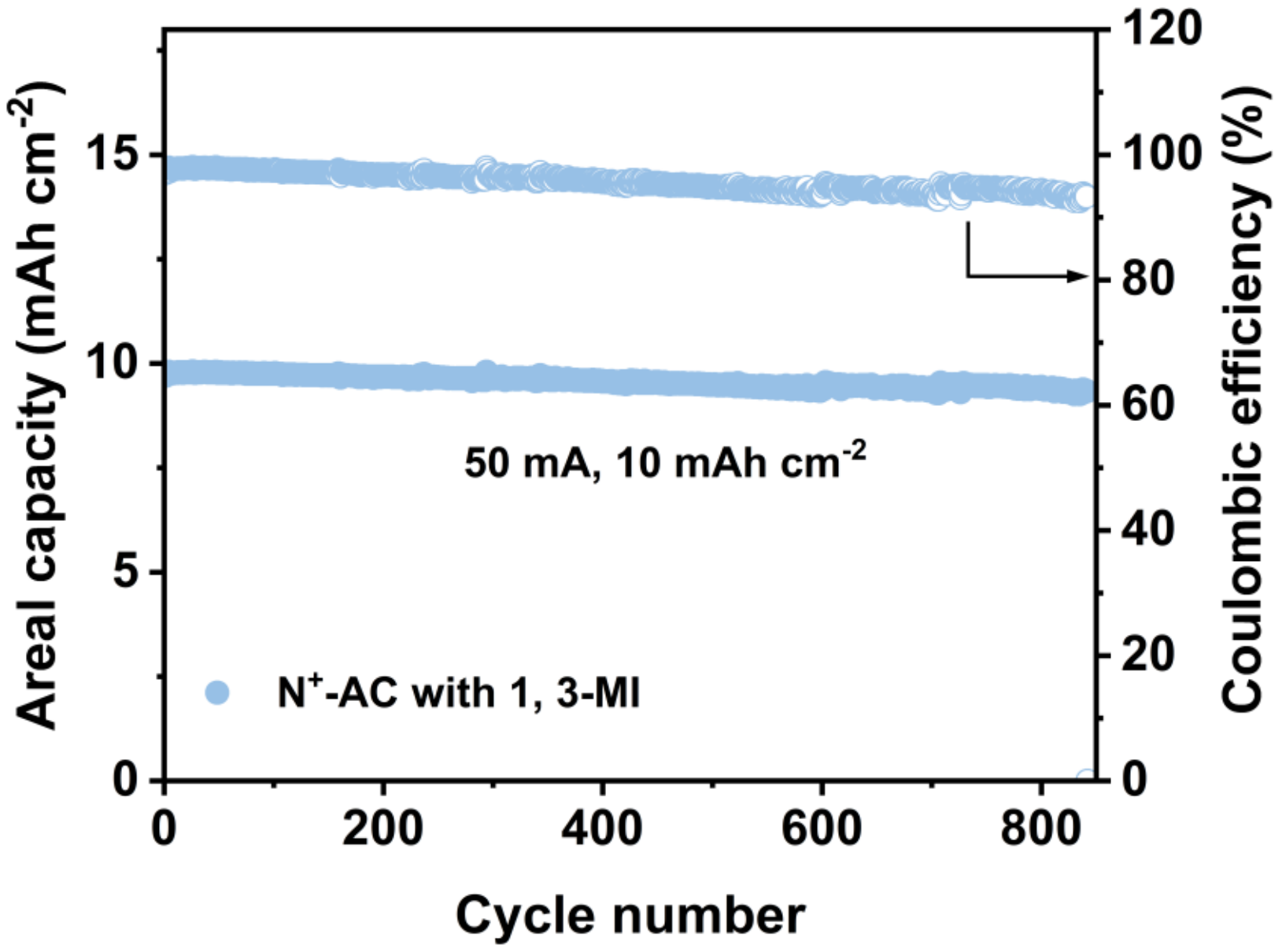

Figure S15. Cycling performance of N^{+}-AC||Zn cell with 1, 3-MI at 50 mA cm^{-2} and 10 mAh cm^{-2}.

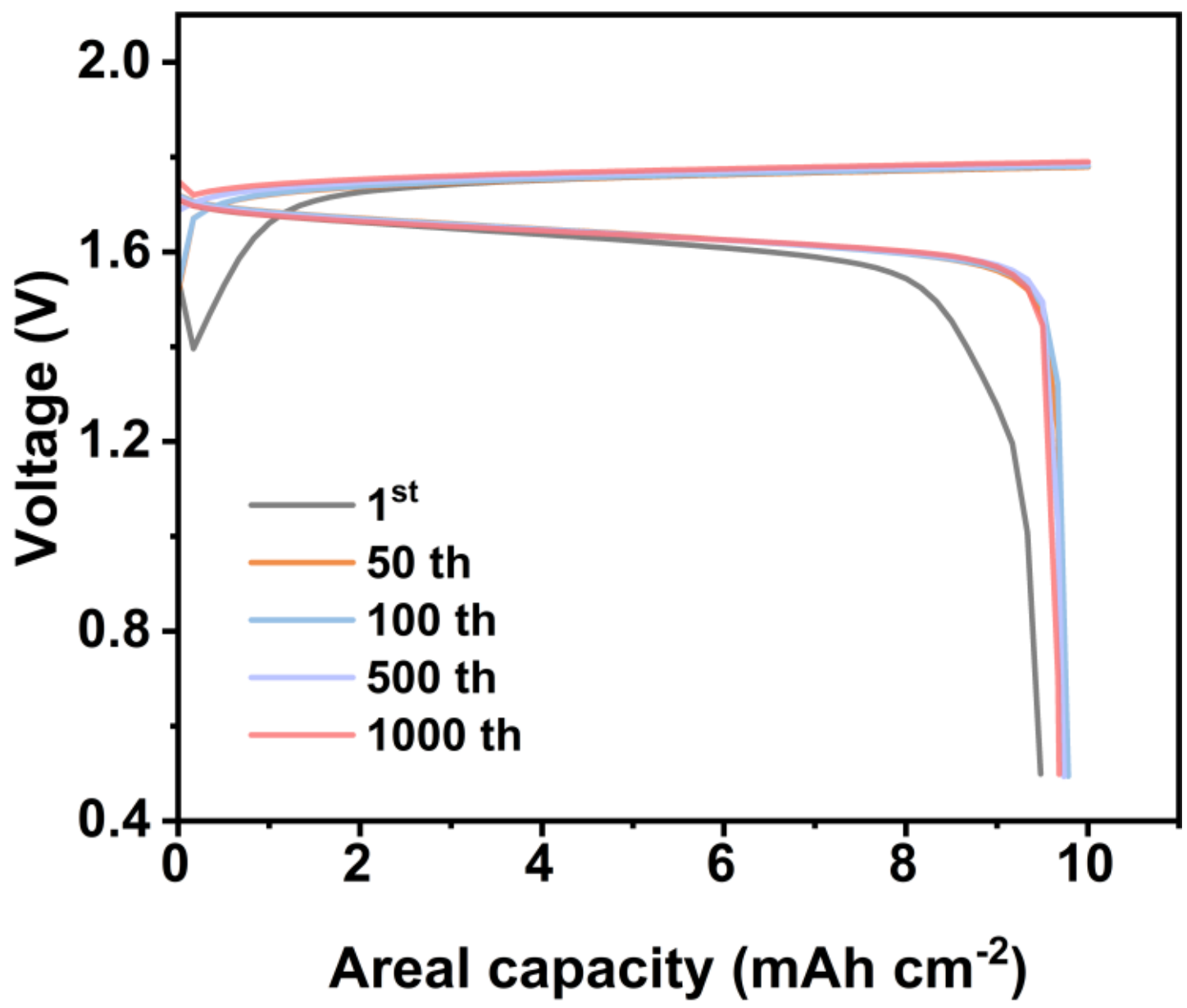

Figure S16. Galvanostatic charge/discharge curves of N^{+}-AC||Ti anode-free cell with 1, 3-MI at 20 mA cm^{-2} and 10 mAh cm^{-2} for different cycles.

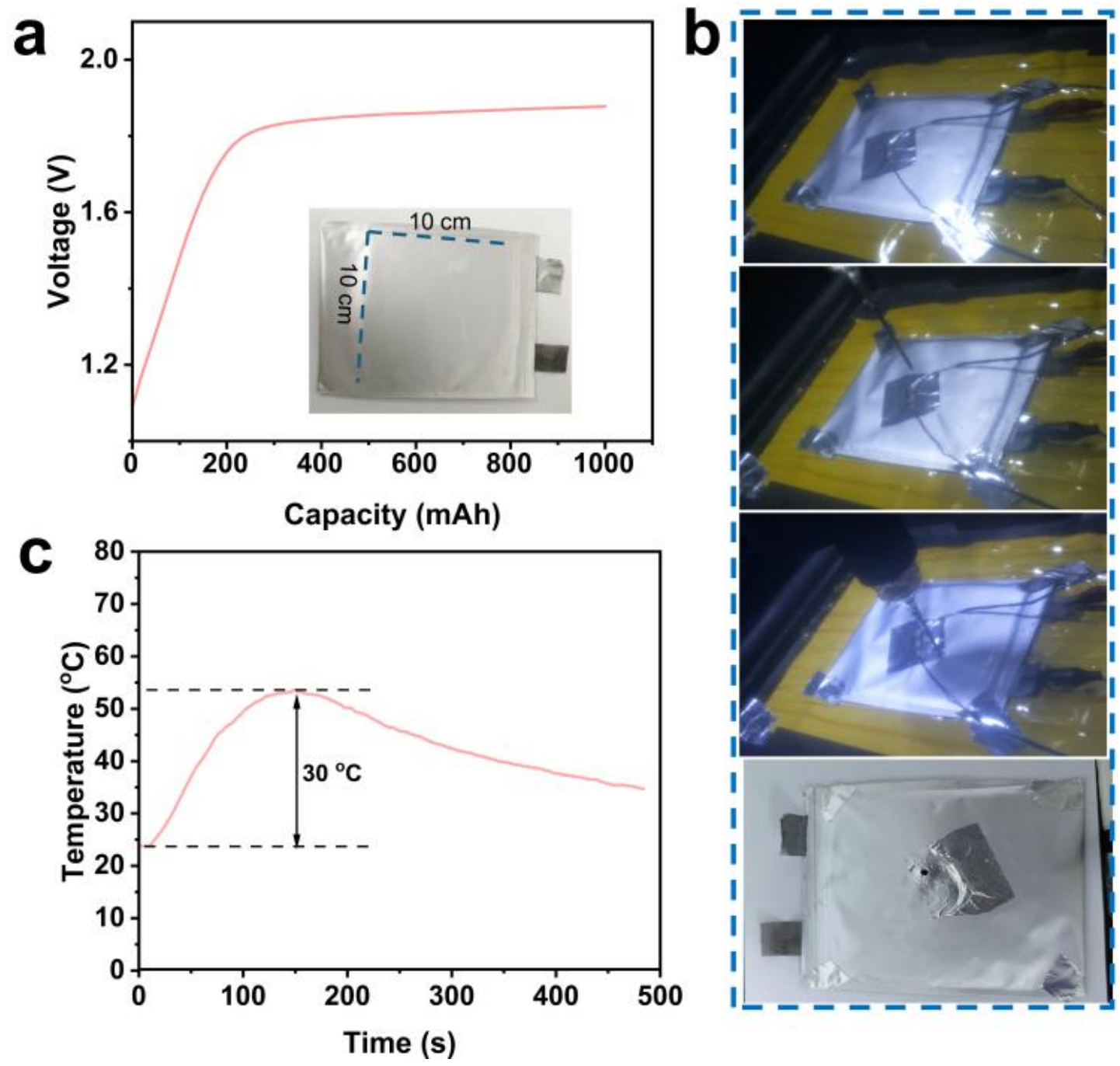

Figure S17. (a) Charging curve of N^{+}-AC||Zn pouch cell with 1, 3-MI (the inset is optical image of the pouch cell). (b) Nail penetration test of the pouch cell. (c) Time-dependent temperature profile under nail penetration.

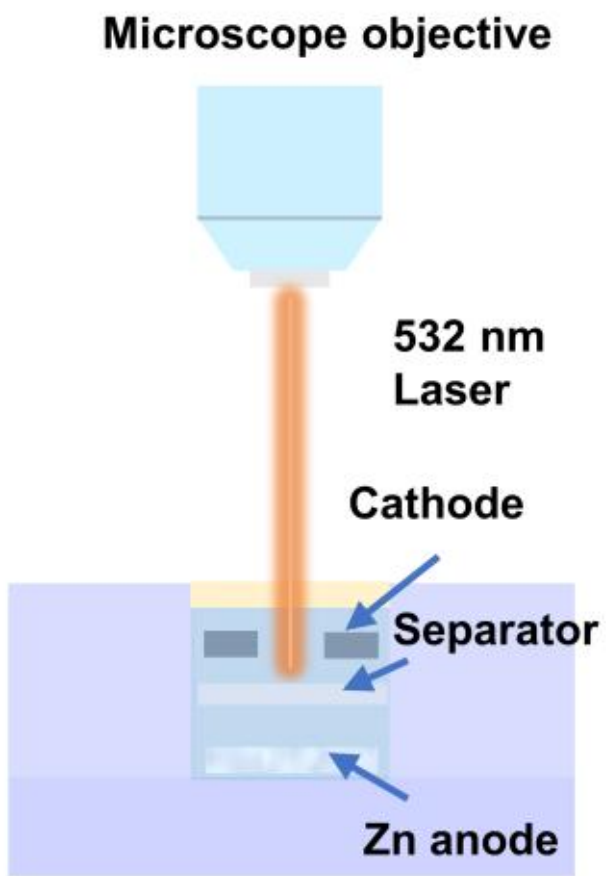

Figure S18. Schematic illustration for the in situ Raman spectroscopy testing of AC||Zn, N^{+}-AC||Zn and N^{+}-AC||Zn cells with 1, 3-MI at room temperature. And the detected region is the electrolyte near the cathode.

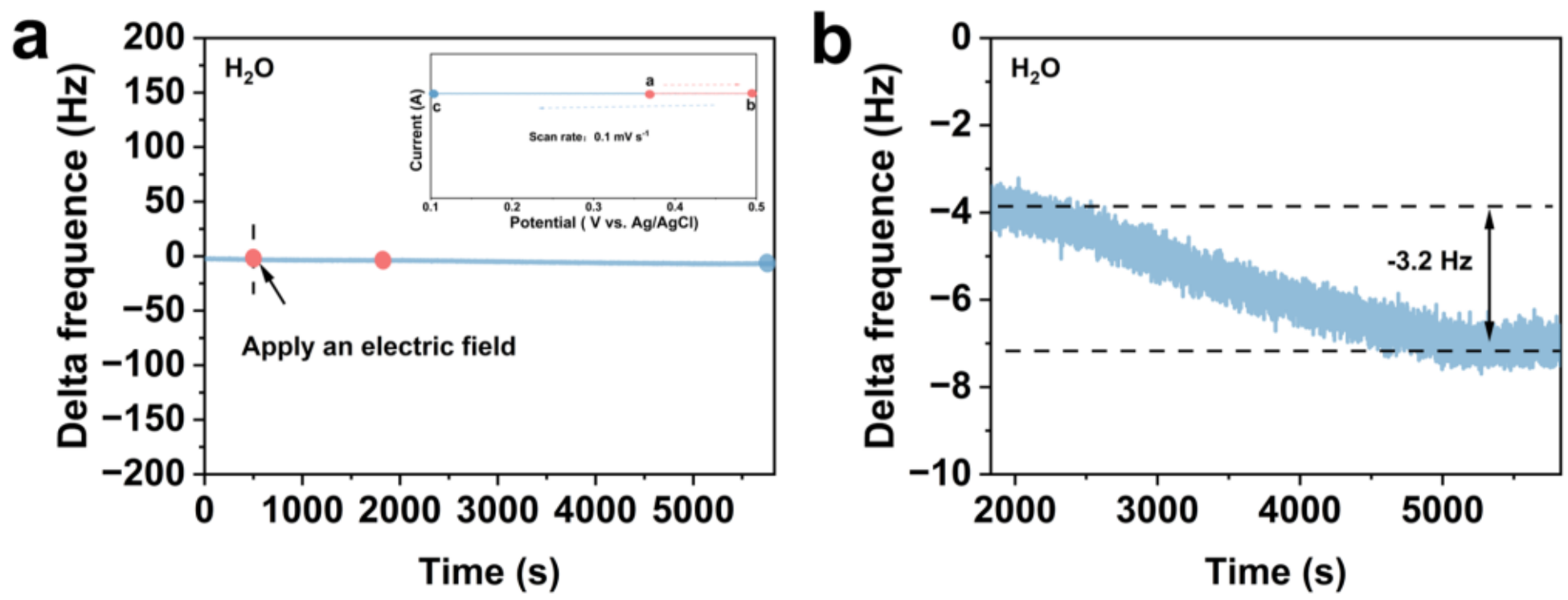

Figure S19. (a) EQCM measurement of pure H_2O. (b) Enlarged plot of frequency-time curve during cathodic scan at 0.1 mV s^{-1}.

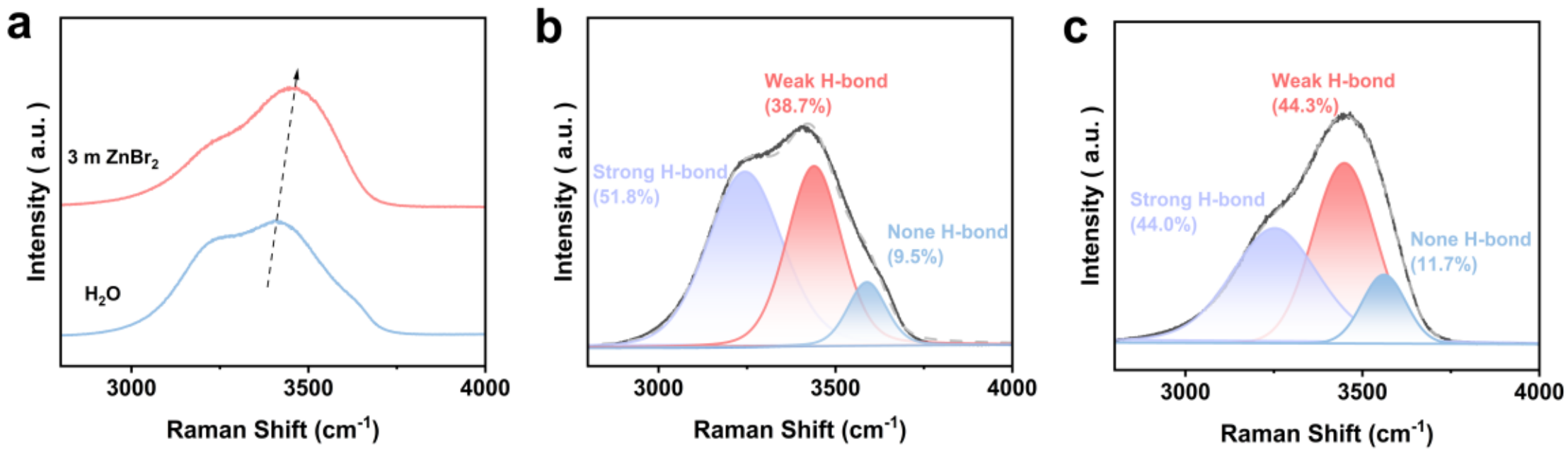

Figure S20. (a) The Raman spectroscopy measurements of H_2O and 3m $ZnBr_2$ electrolyte and (b, c) corresponding peak-fitting spectra.

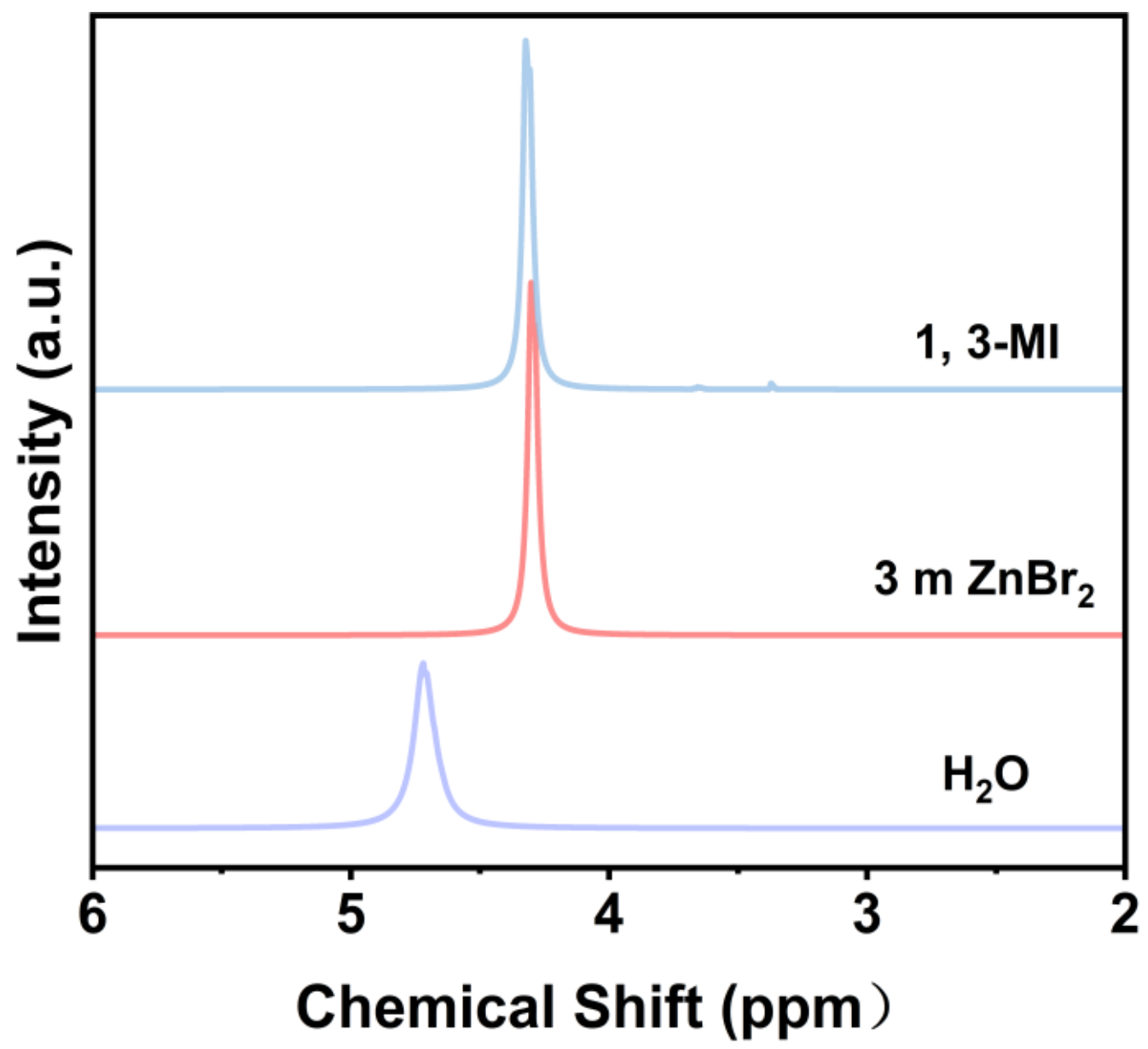

Figure S21. ^{1}H NMR spectra of pure H_2O, 3 m $ZnBr_2$ and 1, 3-MI electrolyte.

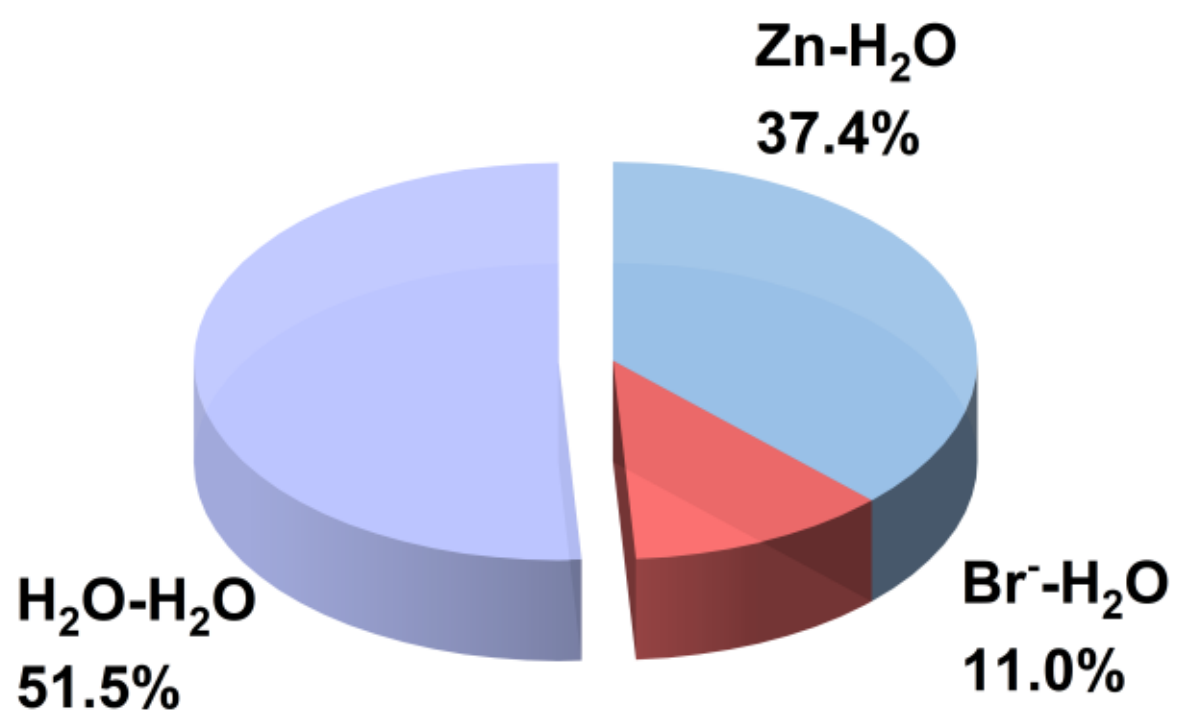

Figure S22. Proportions of H_2O-H_2O, H_2O-Br^-, and H_2O-Zn^{2+} in 3 m $ZnBr_2$ solution.

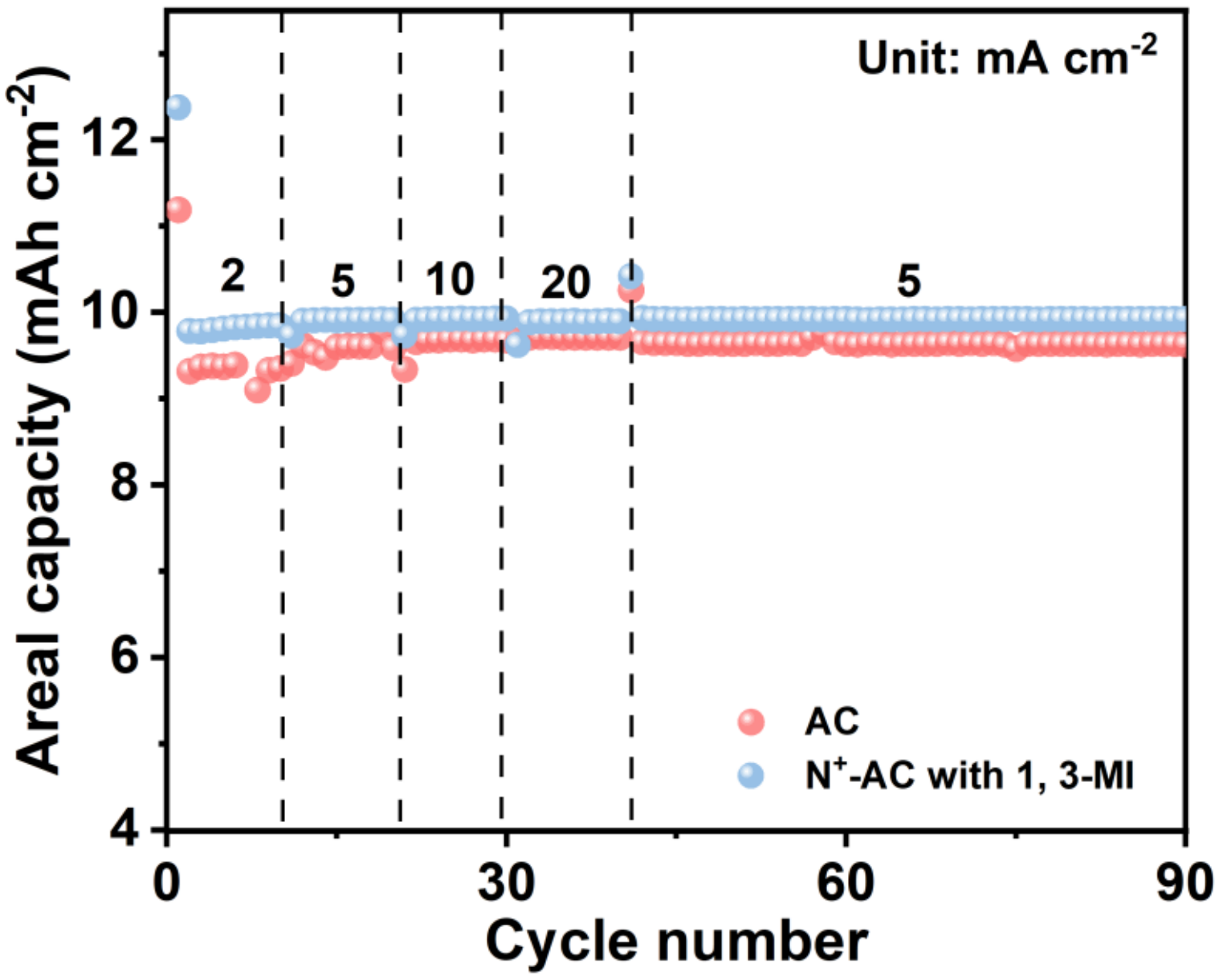

Figure S23. Rate performance of AC||Zn and N^+-AC||Zn cells with 1, 3-MI at -20ºC.

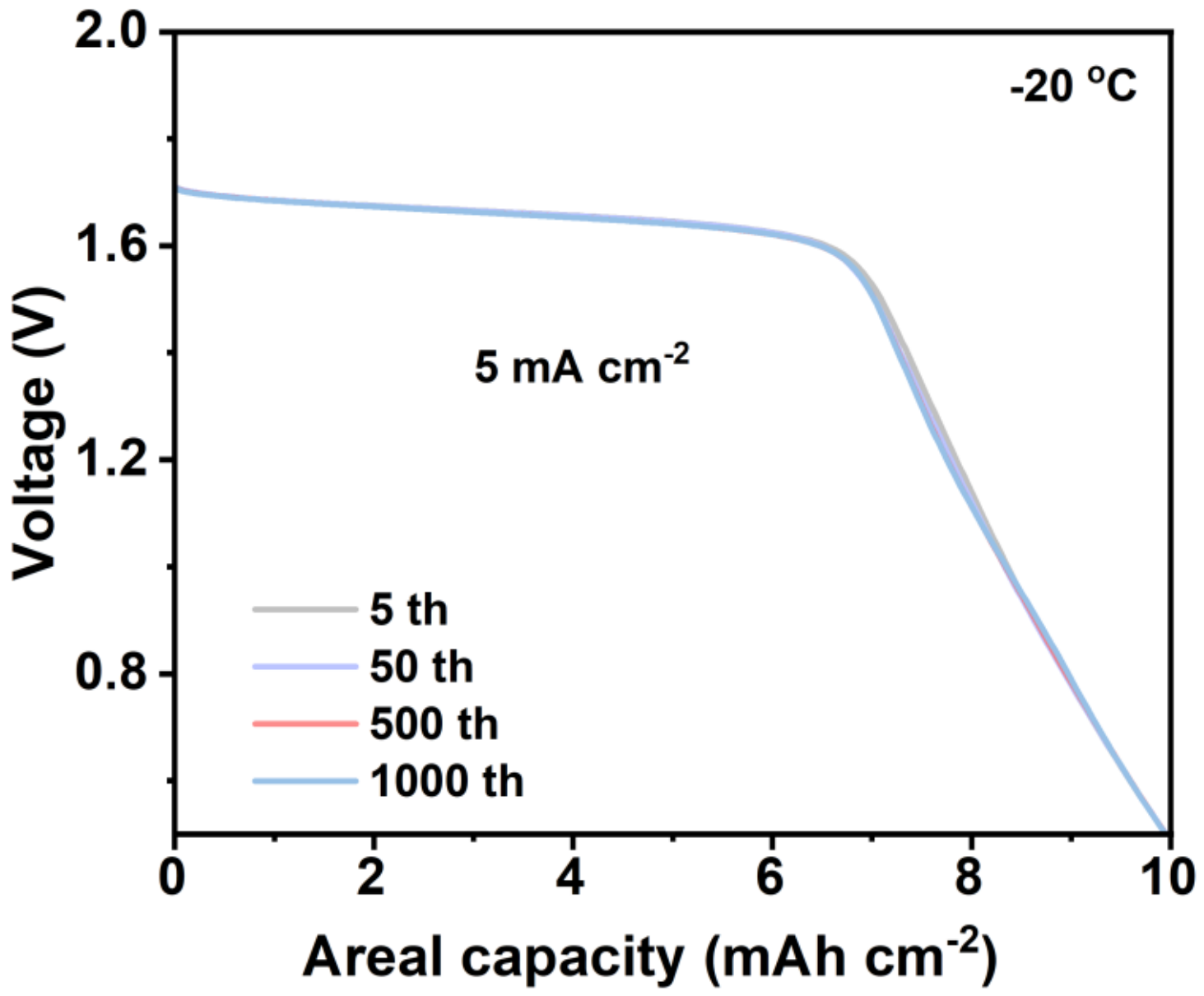

Figure S24. Capacity-voltage curves of N^{+}-AC||Zn cell with 1, 3-MI.

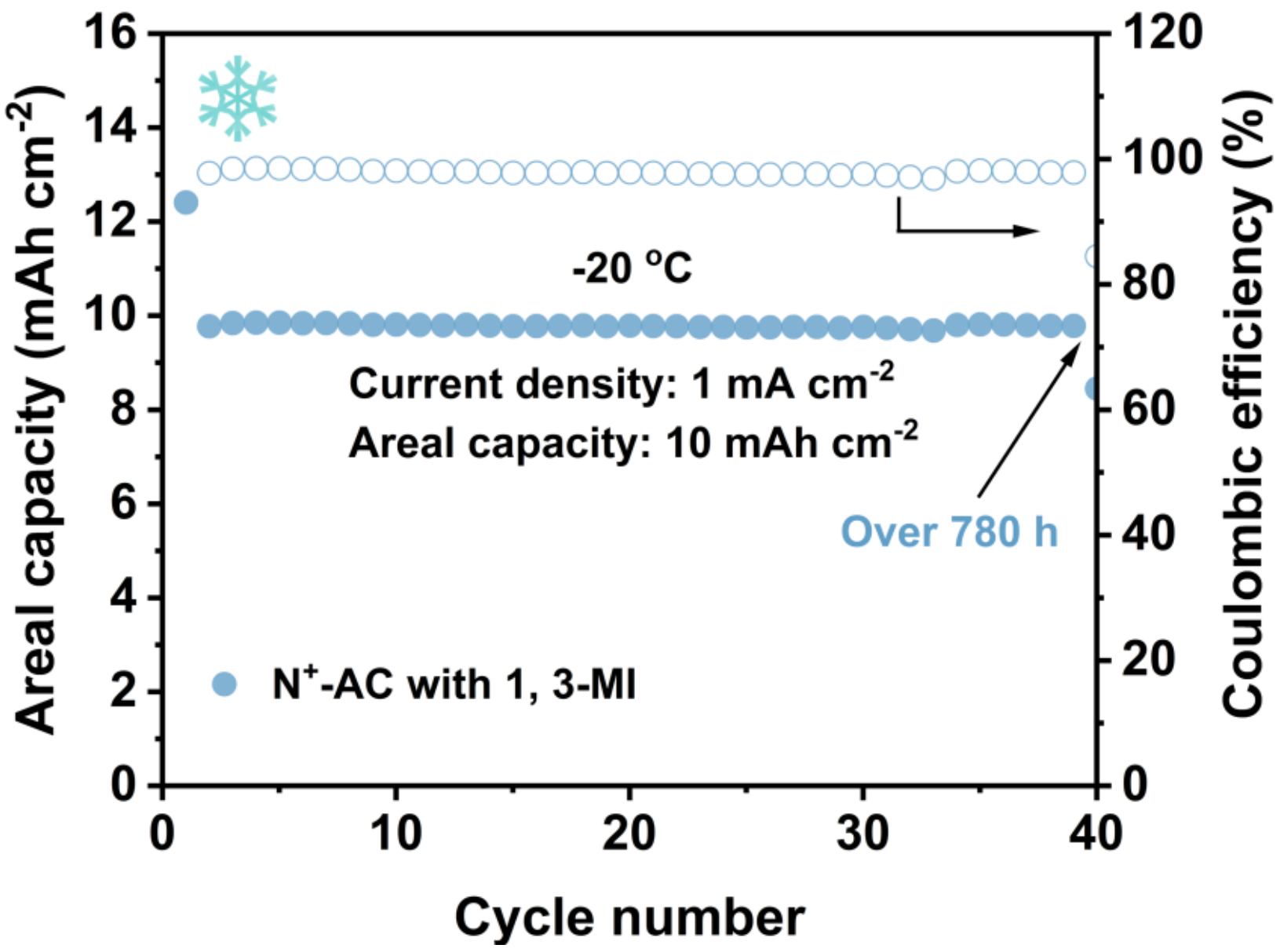

Figure S25. Cycling performance of N^{+}-AC||Zn cell with 1, 3-MI at -20°C under 1 mA cm^{-2} and 10 mAh cm^{-2}.

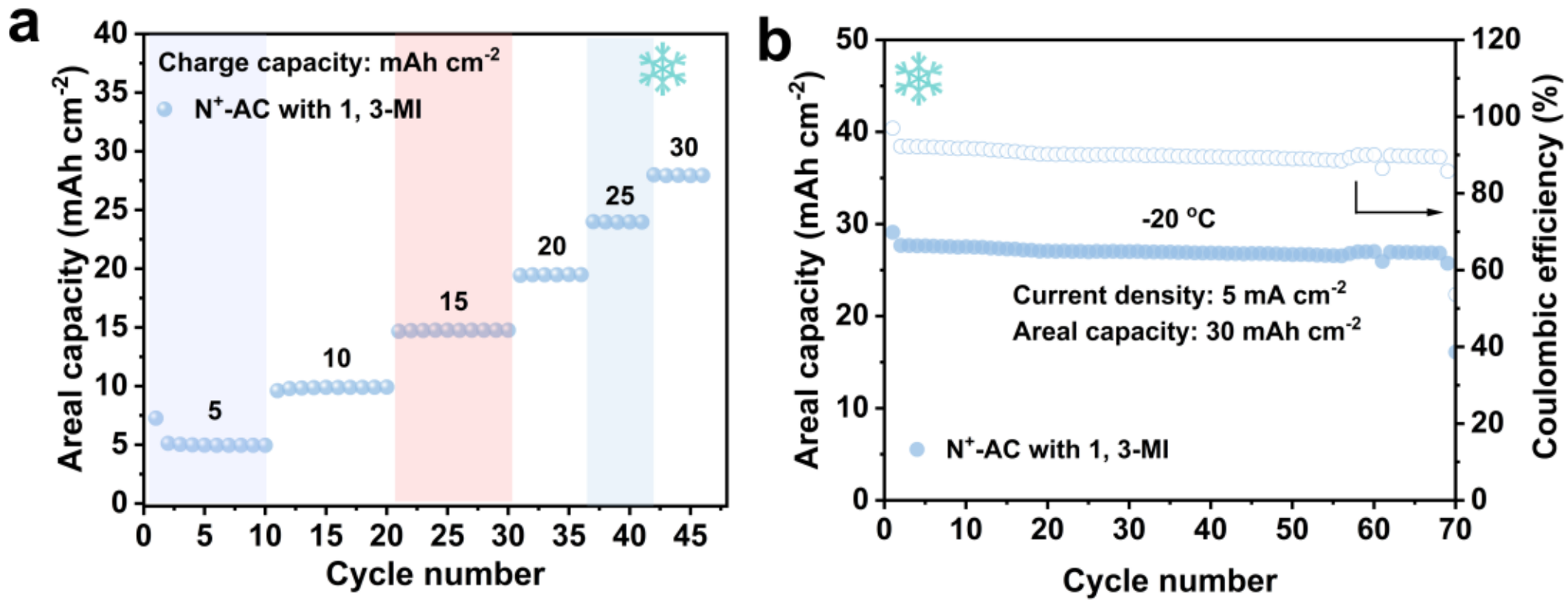

Figure S26. (a) Rate performance of N^{+}-AC||Zn cell with 1, 3-MI charging to different capacity at -20ºC. (b) Cycling performance of N^{+}-AC||Zn cell with 1, 3-MI at -20ºC under 5 mA cm^{-2} and 30 mAh cm^{-2}.

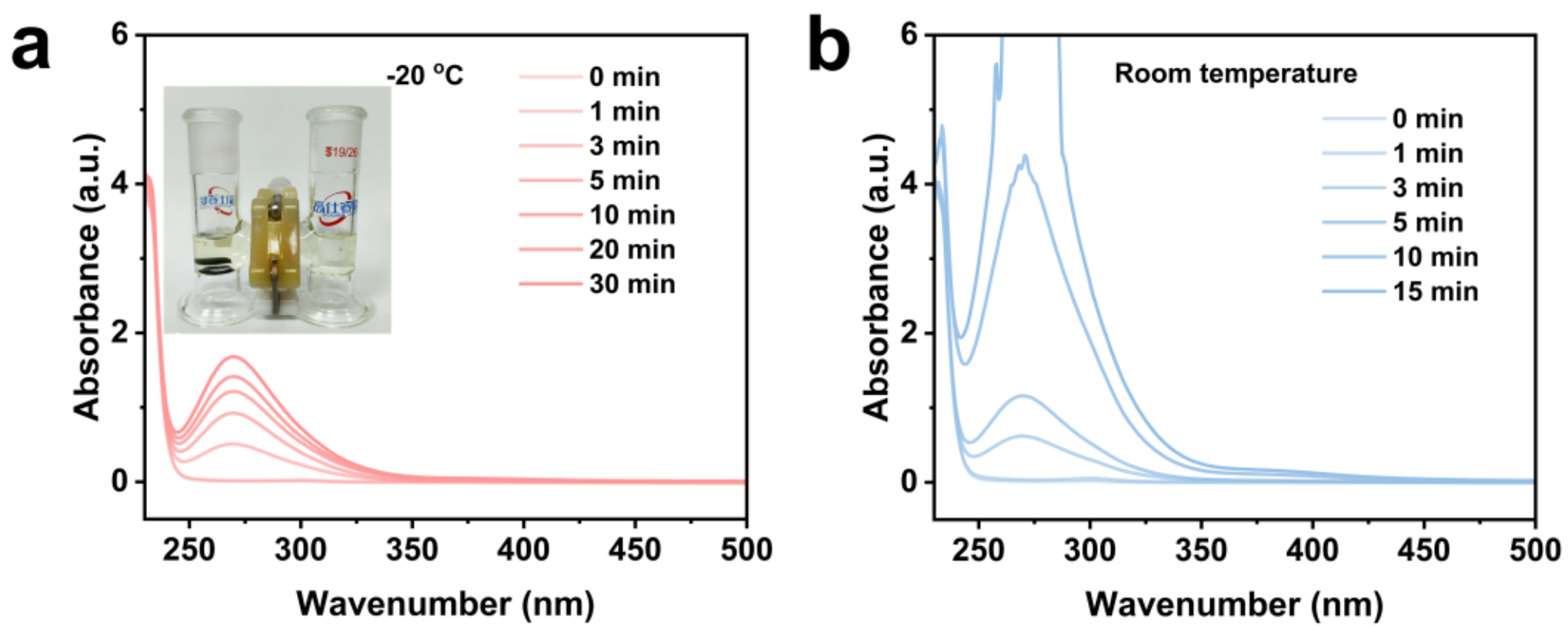

Figure S27. UV-vis spectra of sample 3 m $ZnBr_2$ measured at different time intervals under (a) -20ºC and (b) room temperature conditions.

Note: Specifically, 20 ml 3 m $ZnBr_2$ solution was added to the H-type electrolytic cell. Subsequently, the activated carbon (AC) electrode charged to 10 mAh was placed in one compartment of the H-type cell, and the change in polybromide concentration of the opposite compartment was monitored to characterize the diffusion kinetics of polybromide species. Apparently, the signal intensity of polybromides at -20ºC is significantly lower than that at room temperature, indicating that polybromide diffusion is remarkably suppressed under low-temperature conditions.

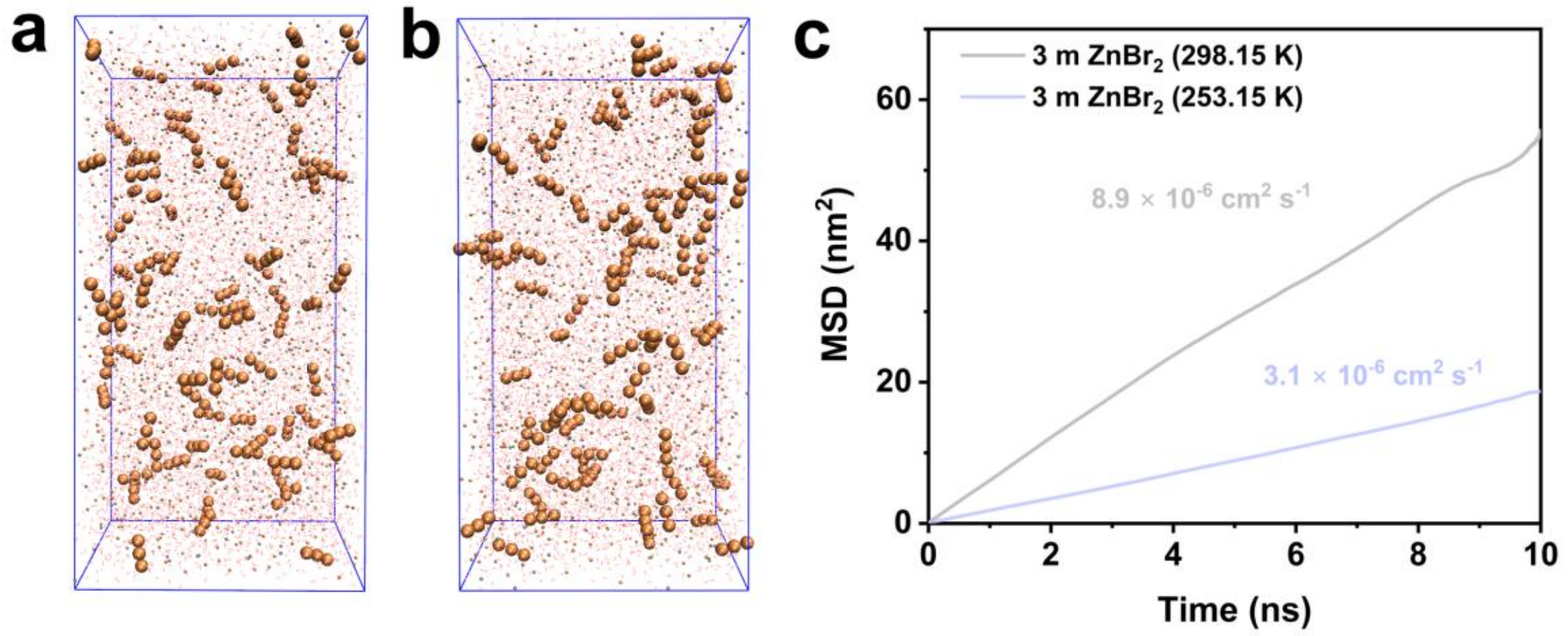

Figure S28. MD simulations of 3 m $ZnBr_2$ electrolyte containing Br_3^- anions at (a) room temperature and (b) -20°C. (c) Mean square displacement (MSD) of Br_3^- anions in 3 m $ZnBr_2$ electrolyte at different temperatures.

Note: Evidently, Br_3^- ions in 3 m $ZnBr_2$ solution exhibit intensified aggregation and reduced diffusion velocity at -20ºC.

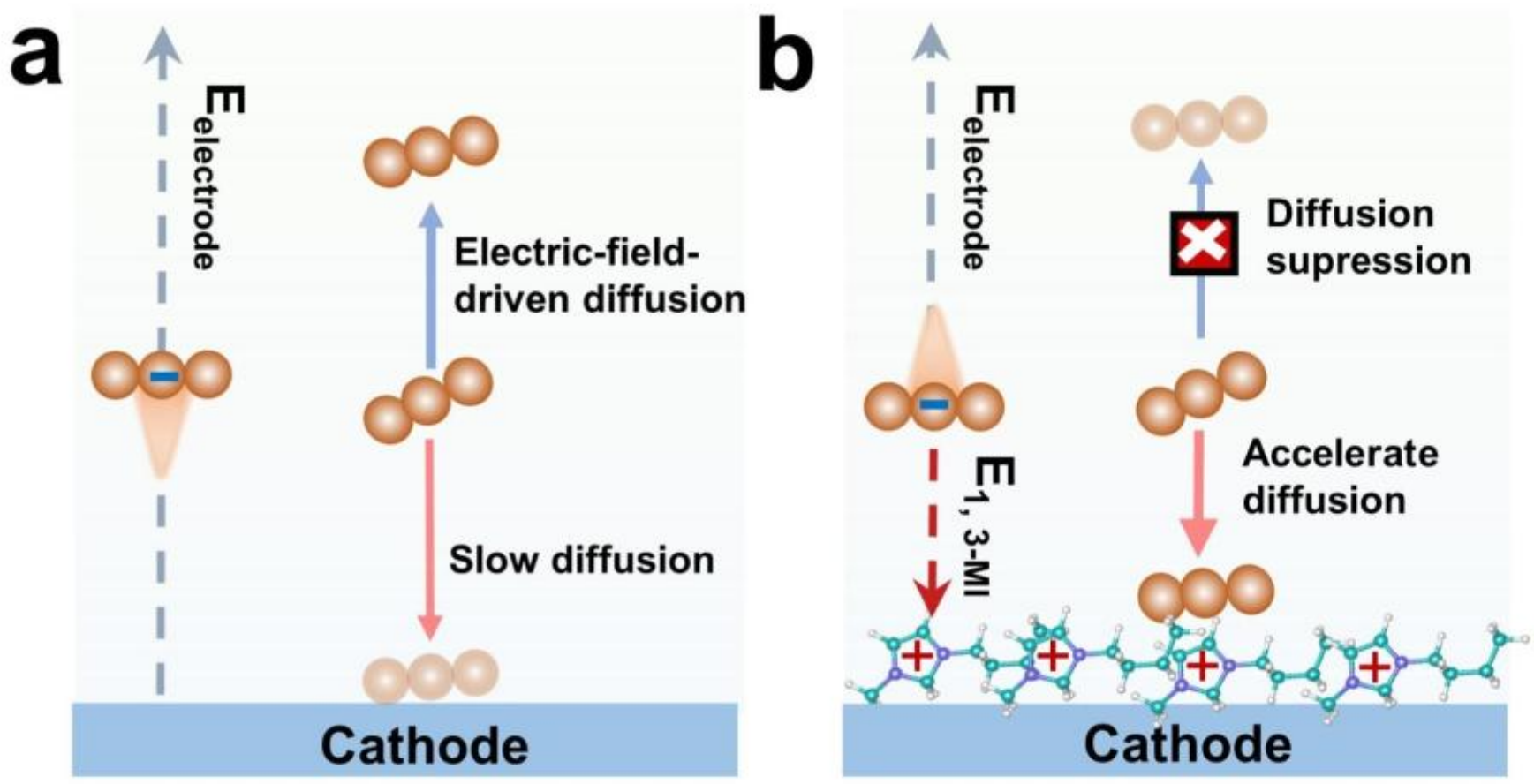

Figure S29. Schematic diagram of Br_3^- migration in (a) CC||Zn with blank electrolyte (10 mM Br_3^- + 3 m $ZnBr_2$) and (b) CC||Zn cell with 1, 3-MI electrolyte containing 10 mM Br_3^- during discharge.

Section 3. Supplementary Tables

Table S1. The electronic conductivity of the AC and N^+-AC powders at the pressure of 4.0 MPa.

Materials	Conductivity, σ (S cm^{-1})
AC	1.65
N^+-AC	1.52

Table S2. Electrochemical performance comparison of the $Zn\text{-}Br_2$ battery at room temperature with previous publications.

Work	Current density	Discharge areal capacity (mAh cm^{-2})	Cycle number	Reference
HPY Br cathode	0.5 C	9.0	100	25
COF-Zn‖Br_2-exCOF	2 A g^{-1}	~0.62	1000	26
SP-PBH composite electrode	8 mA cm^{-2}	1.9	1200	27
DES-ZBB	0.1 mA cm^{-2}	~0.05	900	28
$TmdpPb_2[IBr]_6$ cathode	1.2 A g^{-1}	~0.3-0.4	1000	29
PES/SPEEK@ Zn// N-ACF	10 mA cm^{-2}	1.9	1000	30
ZTE ($TPABr_3$)	0.5 A g^{-1}	~0.2	500	31
Sb@Cu/TPABr-$ZnBr_2$	10 mA cm^{-2}	10.0	800	32
$[BMImB]Br_2$ cathode	0.5 C	10.1	160	33
Zn-Cu@Cu ($TPABr_3$)	10 mA cm^{-2}	5.0	1700	34
LA/$TPABr_3$	0.1 A g^{-1}	5.0	700	35
AP-Cu（TPABr）	5 mA cm^{-2}	5.0	200	36
Gdm_2SO_4/HPYBr-$ZnBr_2$	55 mA g^{-1}	5.0	850	37
**This work**	**20 mA cm^{-2}**	**9.9**<br>**9.7 (anode-free)**	**1000**<br>**2000**	**This work**

Table S3. Electrochemical performance comparison of the $Zn-Br_2$ pouch cell at room temperature with previous publications.

Work	Current density	Capacity (Ah)	Cycle number	Reference
SP-PBH composite electrode	0.1 A	0.018	25	27
Sb@Cu/TPABr-ZnBr2	50 mA	0.4	200	38
[BMImB]Br_2	2 mA cm^{-2}	0.11	100	33
Quasi-solid HPY Br cathode	3 mA cm^{-2}	0.36	200	25
$TPABr_3$ (ZTE and ZW electrolytes)	1 A g^{-1}	~0.006	100	31
BMIMBr and $ZnBr_2$ (SDA)	0.25 C	~1.0	55	39
$TPABr_3$ (Zn–Cu@Cu anode)	200 mA	~1.0	50	40
PAM@$TPABr_3$	\	~1.0	90	41
**This work**	**2 A (10 mA cm^{-2})**	**~1.0**	**145**	**This work**

Table S4. The ionic conductivity of 3 m $ZnBr_2$ and 1, 3-MI electrolyte at room temperature and -20ºC.

Electrolyte	Ionic conductivity (m s^{-1})	
3 m $ZnBr_2$	95. 4 (room temperature)	69.5 (-20 ºC)
1, 3-MI	79.5 (room temperature)	47.5 (-20 ºC)

Table S5. Electrochemical performance comparison of aqueous Zn-based battery systems at -20°C with previous publications.

Work	Cell assembly	Current density	Areal capacity (mAh cm^{-2})	Cycle number	Reference
Pro/$ZnSO_4$ electrolyte (PANI‖Zn)	Coin cell	0.2 A g^{-1}	0.15	260	42
DP additive (Zn-$Zn_{0.58}V_2O_5 \cdot H_2O$)	Coin cell	0.5A g^{-1}	~0.8	500	43
NPA electrolyte (Zn|NPA|ZnVOH)	Coin cell	0.5 A g^{-1} (-15°C)	~0.3	900	44
Zwitterionic pyrrole (ZiPy) additive (Zn‖I_2)	Coin cell	1 A g^{-1}	~0.1-0.2	250	45
EG additive-(Zn‖PANI–V_2O_5)	Coin cell	0.2 A g^{-1}	0.3	< 300	46
WiSE electrolyte (Zn‖$NH_4V_3O_8 \cdot 1.9H_2O$)	Coin cell	0.5 A g^{-1}	~0.1	450	47
AF-gel (Zn‖MnO_2/CNT)	Coin cell	2.4 A g^{-1}	~0.1	600	48
PVA-B-G (Zn‖MnO_2)	Coin cell	1 A g^{-1}	~0.7	2000	49
Mn^{2+}-doped layered vanadium oxide (Zn‖$Mn_{0.15}V_2O_5 \cdot nH_2O$)	Coin cell	3 A g^{-1}	~0.1	3000	50
PAAm hydrogel (Zn‖$LiFePO_4$)	Coin cell	0.5 A g^{-1}	0.08	500	51
PDC-20 gel electrolyte (Zn‖MnO_2/CNT)	Coin cell	0.1 A g^{-1}	0.3	350	52
Cu@AOF (Cu@AOF‖$Zn_{0.5}VO_2$)	Coin cell	1 A g^{-1}	~0.05	2000	53
ZAD4 electrolyte (PANI‖Zn)	Coin cell	0.05 A g^{-1}	1.2	200	54
$ZW_5G_5M_1$ electrolyte (Zn‖VO_2)	Pouch cell	1 A g^{-1}	0.0006 Ah	600	55
BPE electrolyte (Zn‖V_2O_5)	Pouch cell	0.5 A g^{-1}	0.005 Ah	200	56
**This work**	**Coin cell**<br>**Pouch cell**	**5 mA cm^{-2}**<br>**5 mA cm^{-2}**	**10**<br>**~1 Ah**	**1000**<br>**80**	**This work**

Table S6. The viscosity of 3 m $ZnBr_2$ and 1, 3-MI electrolyte at room temperature and -20ºC.

Electrolyte	Viscosity (mPa s^{-1})	
3 m $ZnBr_2$	2.70 (room temperature)	7.94 (-20ºC)
1, 3-MI	2.64 (room temperature)	8.93 (-20ºC)

Section 4. Supplementary References

1. Gao L, Li Z, Zou Y *et al.* A High-Performance Aqueous Zinc-Bromine Static Battery. *iScience* 2020; **23**: 101348.
2. Zhang M,Sun C,Chen G,*et al.* Synergetic bifunctional Cu-In alloy interface enables Ah-level Zn metal pouch cells. *Nat Commun* 2024; **15**: 9455.
3. Frisch, MJ, Trucks GW, Schlegel HB *et al*. Gaussian 09, Revision E.01; Gaussian, Inc., Wallingford CT, 2013.
4. Stephens PJ, Devlin FJ, Chabalowski CF *et al.* Ab Initio Calculation of Vibrational Absorption and Circular Dichroism Spectra Using Density Functional Force Fields. *J Phys Chem* 1994; **98**: 11623–11627.
5. Stephens PJ, Jalkanen KJ, Devlin FJ *et al.* Ab initio calculation of vibrational circular dichroism spectra using accurate post-self-consistent-field force fields: trans-2,3-dideuteriooxirane. *J Phys Chem* 1993; **97**: 6107–6110.
6. Weigend F, Ahlrichs R. Balanced basis sets of split valence, triple zeta valence and quadruple zeta valence quality for H to Rn: Design and assessment of accuracy. *Phys. Chem Chem Phys* 2005; **7**: 3297–3305.
7. Weigend F. Accurate Coulomb-fitting basis sets for H to Rn. *Phys Chem Chem Phys* 2006; **8**: 1057–1065.
8. Marenich AV, Cramer CJ, Truhlar DG. Universal Solvation Model Based on Solute Electron Density and on a Continuum Model of the Solvent Defined by the Bulk Dielectric Constant and Atomic Surface Tensions. *J Phys Chem B* 2009; **113**: 6378–6396.
9. Bayly CI, Cieplak P, Cornell W *et al.* A well-behaved electrostatic potential based method using charge restraints for deriving atomic charges: the RESP model. *J Phys Chem* 1993; **97**: 10269–10280.

10. Lu T, Chen F. Multiwfn: A multifunctional wavefunction analyzer. *J Comput Chem* 2012; **33**: 580–592.
11. Zhang J, Lu T. Efficient evaluation of electrostatic potential with computerized optimized code. *Phys Chem Chem Phys* 2021; **23**: 20323–20328.
12. Kresse G, Furthmüller J. Efficient iterative schemes for ab initio total-energy calculations using a plane-wave basis set. *Phys Rev B* 1996; **54**: 11169–11186.
13. Humphrey W, Dalke A, Schulten K. VMD: Visual molecular dynamics. *J Mol Graph* 1996; **14**: 33–38.
14. Grimme S, Ehrlich S, Goerigk L. Effect of the damping function in dispersion corrected density functional theory. *J Comput Chem* 2011; **32**: 1456–1465.
15. Blöchl P, E,. Projector augmented-wave method. *Phys Rev B* 1994; **50**: 17953–17979.
16. Grimme S, Antony J, Ehrlich S *et al.* A consistent and accurate ab initio parametrization of density functional dispersion correction (DFT-D) for the 94 elements H-Pu. *J Chem Phy* 2010.; **132**: 154104.
17. Wang V, Xu N, Liu J *et al.* VASPKIT: A user-friendly interface facilitating high-throughput computing and analysis using VASP code. *Comput Phys Commun* 2021; **267**: 108033.
18. Abraham MJ, Murtola T, Schulz R *et al.* GROMACS: High performance molecular simulations through multi-level parallelism from laptops to supercomputers. *SoftwareX* 2015; **1–2**: 19–25.
19. Berendsen HJC, Grigera J R, Straatsma TP. The missing term in effective pair potentials. *J Phys Chem* 1987; **91**: 6269–6271.
20. Lindorff-Larsen K, Piana S, Palmo K *et al.* Improved side-chain torsion potentials for the Amber ff99SB protein force field. *Proteins Struct Funct. Bioinforma* 2010; **78**: 1950–1958.
21. Lu, T. Sobtop, Version [1.0], http://sobereva.com/soft/Sobtop: 2022.
22. Kohn W, Sham LJ. Self-Consistent Equations Including Exchange and Correlation Effects. *Phys Rev* 1965; **140**: A1133–A1138.
23. Banerjee A, Adams N, Simons J *et al.* Search for stationary points on surfaces. *J Phys Chem* 1985; **89**: 52–57.

24. Martínez L, Andrade R, Birgin EG *et al.* PACKMOL: A package for building initial configurations for molecular dynamics simulations. *J Comput Chem* 2009; **30**: 2157–2164.
25. Xu C, Lei C, Jiang P *et al.* Practical high-energy aqueous zinc-bromine static batteries enabled by synergistic exclusion-complexation chemistry. *Joule* 2024; **8**: 461–481.
26. Zhang Y, Wei C, Wu M *et al.* A high-performance COF-based aqueous zinc-bromine battery. *Chem Eng J* 2023; **451**: 138915.
27. Wu W, Xu S, Lin Z *et al.* A polybromide confiner with selective bromide conduction for high performance aqueous zinc-bromine batteries. *Energy Storage Mater* 2022; **49**: 11–18.
28. Heo J, Shin K, Kim H. A Zinc–Bromine Battery with Deep Eutectic Electrolytes. *Adv Sci* 2022; **9**: 2204908.
29. Wang S, Wang Y, Wei Z *et al.* Halide Exchange in Perovskites Enables Bromine/Iodine Hybrid Cathodes for Highly Durable Zinc Ion Batteries. *Adv Mater* 2024; **36**: 2401924.
30. Liu C, Dong W, Zhou H *et al.* Achievement of Efficient and Stable Nonflow Zinc–Bromine Batteries Assisted by Rational Decoration upon the Two Electrodes. *ACS Appl Mater Interfaces* 2024; **16**: 23278–23287.
31. Li W, Xu H, Ke S *et al.* Integrating Electric Ambipolar Effect for High-Performance Zinc Bromide Batteries. *Nano-Micro Lett* 2025; **17**: 143.
32. Sun J, Zheng X, Zhu Z *et al.* Engineering Artificial Protrusions of Zn Anodes for Aqueous Zinc Batteries. *Nano Lett* 2025; **25**: 7266–7275.
33. Wu Y, Xu C, Lei C *et al.* Optimizing Bromine Complexation and Kinetics: a Bisimidazole Strategy for High-Performance Zn-Br Static Batteries. *Angew Chem Int Ed* 2025; **64**: e202509293.
34. Han B, Zheng X, Liu S *et al.* Anode-Free Zinc–Bromine Batteries Enabled by a Simple Prenucleation Strategy. *Small* 2025; **21**: 2408982.
35. Shi M, Lei C, Wang H *et al.* Molecule Engineering of Sugar Derivatives as Electrolyte Additives for Deep-Reversible Zn Metal Anode. *Angew Chem Int Ed* 2024; **63**: e202407261.
36. Zheng X, Liu Z, Sun J *et al.* Constructing robust heterostructured interface for anode-free zinc batteries with ultrahigh capacities. *Nat Commun* 2023; **14**: 76.

37. Xu C, Wang H, Lei C *et al.* Fast single metal cation conduction in ion-water aggregated aqueous battery electrolytes. *Nat Commun* 2025; **16**: 4574.
38. Zheng X, Luo R, Liu Z *et al.* A practical zinc-bromine pouch cell enabled by electrolyte dynamic stabilizer. *Mater Today* 2024; **80**: 353–364.
39. Luo R, Zheng X, Jiang T *et al.* Reshaping Electrical Double Layer via Synergistic Dual Additives for Ah-Level Zinc Battery. *Adv Energy Mater* 2025; **15**: 2501658.
40. Han B, Zheng X, Liu S *et al.* Anode-Free Zinc–Bromine Batteries Enabled by a Simple Prenucleation Strategy. *Small* 2025; doi:10.1002/smll.202408982.
41. Zheng X, Wu S, Luo R *et al.* An Inter-Locking Quasi-Solid Cathode for Zinc-Bromine Batteries with Stable 32000 Cycles. *Adv Mater* 2025; 10.1002/adma.202517292.
42. Xing Z, Ye P, Shi X *et al.* Design of Cryogenic Electrolyte with Organic-Free Solvation Structure for Wide-Temperature Zinc Metal Batteries. *Angew Chem Int Ed* 2025; **64**: e202516974.
43. Zhang Y, Zhang Z, Dou H *et al.* Engineering Nanoscale Interfacial Solvation Inner-Outer Configuration via Multi-Group Synergy for Practical Zinc Batteries. *Angew Chem Int Ed* 2025; doi: 10.1002/adma.202517292.
44. Liu Z, Sun J, Li X *et al.* Constructing eutectic solvation sheath by weak solvation effect for stabilizing Zn-ion batteries with low-temperature adaptability. *Energy Storage Mater* 2025; **83**: 104727.
45. Wang S, Chen Y, Donkor S *et al.* Zwitterionic Electrolyte Additives Empowered Robust Zn–I_2 Batteries Enduring a Low Temperature of −40 °C. *Adv Sci* 2025; doi: 10.1002/advs.202518135
46. Chang N, Li T, Li R *et al.* An aqueous hybrid electrolyte for low-temperature zinc-based energy storage devices. *Energy Environ Sci* 2020; **13**: 3527–3535.
47. Chen Y, Zhao J, Wang Y, Quasi-Solid-State Zinc Ion Rechargeable Batteries for Subzero Temperature Applications. *ACS Appl Energy Mater* 2020; **3**: 9058–9065.
48. Mo F, Liang G, Meng Q *et al.* A flexible rechargeable aqueous zinc manganese-dioxide battery working at −20 °C. *Energy Environ Sci* 2019; **12**: 706–715.
49. Chen M, Zhou W, Wang A *et al.* Anti-freezing flexible aqueous Zn–MnO_2 batteries working at −35 °C enabled by a borax-crosslinked polyvinyl alcohol/glycerol gel electrolyte. *J Mater Chem A* 2020; **8**: 6828–6841.

50. Geng H, Cheng M, Wang B *et al.* Electronic Structure Regulation of Layered Vanadium Oxide via Interlayer Doping Strategy toward Superior High-Rate and Low-Temperature Zinc-Ion Batteries. *Adv Funct Mater* 2020; **30**: 1907684.
51. Zhu M, Wang X, Tang H *et al.* Antifreezing Hydrogel with High Zinc Reversibility for Flexible and Durable Aqueous Batteries by Cooperative Hydrated Cations. *Adv Funct Mater* 2020; **30**: 1907218.
52. Huang S, He S, Li Y *et al.* Hydrogen bond acceptor lined hydrogel electrolyte toward Dendrite-Free aqueous Zn ion batteries with low temperature adaptability. *Chem Eng J* 2023; **464**: 142607.
53. Wang C, Wang D, Lv D *et al.* Interface Engineering by Hydrophilic and Zincophilic Aluminum Hydroxide Fluoride for Anode-Free Zinc Metal Batteries at Low Temperature. *Adv Energy Mater* 2023; **13**: 2204388.
54. Geng L, Meng J, Wang X *et al.* Organic-solvent-free primary solvation shell for low-temperature aqueous zinc batteries. *Chem* 2025; **11**: 102302.
55. Wei X, Guan J, Mu Y *et al.* Decoding Hydrogen-Bond Network of Electrolyte for Cryogenic Durable Aqueous Zinc-Ion Batteries. *Nano-Micro Lett 2026*; **18**: 127.
56. Xu M, Zhang B, Sang Y *et al.* Bicontinuous-phase electrolyte for a highly reversible Zn metal anode working at ultralow temperature. *Energy Environ Sci* 2024; **17**: 8966–8977.
